# A Novel Fluorescence Probe Based on Azamonardine for Detecting and Imaging Cysteine in Cells and Zebrafish with High Selectivity and Sensitivity

**DOI:** 10.3390/molecules28176246

**Published:** 2023-08-25

**Authors:** Yixu Zhao, Ting Wang, Ahmed Mohammed Ali Abdulkhaleq, Zhongfu Zuo, Yongjin Peng, Xibin Zhou

**Affiliations:** 1 Department of Anatomy, Histology and Embryology, Jinzhou Medical University, Jinzhou 121001, China; zhaoyx@stu.jzmu.edu.cn; 2 Liaoning Key Laboratory of Diabetic Cognitive and Perceptive Dysfunction, Jinzhou Medical University, Jinzhou 121001, China; 3 Faculty of Life Sciences and Technology, Kunming University of Science and Technology, Kunming 650500, China; wangt@stu.jzmu.edu.cn; 4 College of Pharmacy, Jinzhou Medical University, Jinzhou 121001, China; hamd@stu.jzmu.edu.cn; 5 College of Basic Science, Jinzhou Medical University, Jinzhou 121001, China

**Keywords:** cysteine, fluorescent probe, azamonardine, cellular imaging

## Abstract

A novel fluorescent probe based on azamonardine (Aza) fluorophore was designed and synthesized for the highly selective detection of cysteine (Cys) in vivo and in vitro. After reacting with acryloyl chloride, the fluorescence of Aza is effectively quenched, resulting in the formation of the Aza-acryl probe. Upon the addition of Cys, the ester bond of Aza-acryl is cleaved, releasing a new compound (Compound **1**) with strong fluorescence, thereby achieving fluorescence turn-on detection of Cys. The structure of Aza-acryl was characterized using X-ray crystallography and NMR spectroscopy. Additionally, density functional theory was employed to elucidate the quenching mechanism of the acyl group on the Aza. Aza-acryl exhibits high selectivity towards Cys and distinguishes it from other biothiols such as homocysteine (Hcy) and glutathione (GSH). The mechanism of Aza-acryl for detecting Cys was investigated through HPLC, NMR spectroscopy, high-resolution mass spectrometry, and reaction kinetics experiments. Aza-acryl demonstrates excellent imaging capabilities for Cys in cells and zebrafish, providing a reliable and selectable tool for the detection and imaging of Cys in biological systems.

## 1. Introduction

Biothiols such as cysteine (Cys), homocysteine (Hcy), and glutathione (GSH) play a crucial role in various biological reactions [1,2]. Cysteine, in particular, is an intrinsically nucleophilic amino acid found in proteins that adjust various biochemical functions [3]. Moreover, it plays a vital role in physiological and pathological functions such as detoxification and metabolism [4,5]. It functions as a source of protein that is beneficial to the human metabolism when at normal levels of 30–200 μM [6]. Abnormal levels of cysteine have been linked to various disorders such as edema [7], liver damage [8], skin lesions [9], Huntington’s disease [10] and Alzheimer’s disease [11]. Therefore, accurately identifying and determining cysteine levels in living systems is vital in monitoring biological metabolism, maintaining the immune system, preventing tissue/DNA damage, preventing autoimmune diseases, and diagnosing relevant disorders.

Various analytical methods are available for the detection of biothiols, including gas chromatography [12], high-performance liquid chromatography [13,14], mass spectrometry [15], and the electrochemical method [16]. However, these methods are expensive, time consuming, or require specialized personnel [17]. Therefore, simple, accurate, and low-cost techniques are necessary for detecting these biomolecules. Fluorescent probes, due to their high sensitivity, selectivity, rapid response, and simplicity, have become a valuable tool for detecting biothiols [18,19,20]. These probes operate through different mechanisms such as condensation with cyanogroups [21], nucleophilic substitution and rearrangement [22], cyclization with aldehyde [23], conjugate addition to maleimide [24], and aromatic substitution rearrangement [25]. Due to their structural similarity, distinguishing Cys, Hcy, and GSH under identical conditions using fluorescent probes is a challenge [26,27,28]. Fluorescent probes based on acryloyl-group-linked fluorophores have gained widespread attention due to their ability to selectively identify Cys. The fluorophores utilized by these fluorescent probes include rhodamine [29], coumarin [30], naphthalimide [31], isophorone [32,33], and carbon dots [34]. 

The azamonardine (Aza) fluorescent group was synthesized and extensively studied in 2013 by the research group led by Francisco Amat-Guerri [35]. It possesses several advantages, including high quantum yield, photostability, and biocompatibility. In recent years, fluorescence probes based on the Aza fluorescent group have been used for the detection of various substances. Researchers often employ different strategies or mechanisms when designing fluorescent probes based on Aza [36]. The generation of Aza requires four conditions: dopamine, catechol, oxygen, and alkalinity. In the presence of three of these conditions, the strong fluorescent Aza can be generated for the detection of dopamine [37,38,39,40], catechol [41,42], oxygen, or volatile alkaline amines [43]. As tyrosinase can convert monophenolamine into dopamine, this strategy can also be used for the detection of tyrosinase or alkaline phosphatase [44,45,46,47,48]. Furthermore, the quantum yield of Aza is closely related to pH, allowing it to be used for the detection of acidic or alkaline gases [49]. Additionally, the internal filtering effect of quantum dots or Cr^6+^ ions on Aza can be utilized in the development of sensing platforms for the detection of biothiols [50], chromium ions, or ascorbic acid [51]. Moreover, the complexation of functionalized catechol derivatives with heavy metal ions can result in the quenching of azamonardine fluorescence, enabling the detection of Cu^2+^ and Hg^2+^ ions [52,53]. 

In this study, a novel fluorescence probe for the detection of Cys was designed and developed based on the Aza fluorescent group. The fluorescence probe was obtained through the simultaneous reaction of the phenolic hydroxyl and alcoholic hydroxyl groups of Aza with acryloyl chloride. During the reaction, the double bond in the acryloyl ester undergoes Michael addition with the amino group of Aza, resulting in the formation of a new structure-based fluorescent probe. The structure was confirmed using NMR spectroscopy, MS, and X-ray crystallography. Upon the addition of Cys, the ester bonds of Aza-acryl broke, and a new compound (Compound **1**) with strong fluorescence was released. The probe exhibits high selectivity and sensitivity and rapid detection of Cys, and was successfully applied for Cys detection in cells and zebrafish. 

## 2. Results and Discussion

### 2.1. Synthesis and Design of Aza-acryl

**Figure 1:**
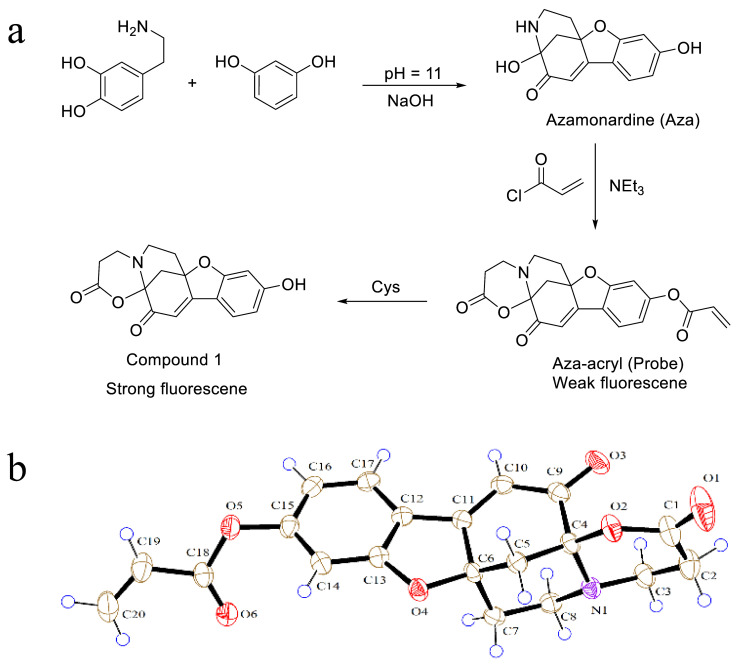
(**a**) Synthesis and design of Aza-acryl (the probe); (**b**) crystal structure of Aza-acryl.

As shown in Figure 1a, the synthesis of Aza-acryl is conducted in two steps. First, dopamine and hydroquinone react under alkaline conditions to generate Aza, a compound with bright fluorescence. Then, Aza reacts with acryloyl chloride to form the probe Aza-acryl. The single crystal structure of Aza-acryl is shown in Figure 1b. From the structure of Aza-acryl, it is evident that both the phenolic hydroxyl and alcoholic hydroxyl of Aza react with acryloyl chloride. During the reaction, the secondary amino group originally present in the Aza structure undergoes Michael addition with the double bond of the acryloyl group on the alcoholic oxygen atom, resulting in the formation of a stable polycyclic compound, Aza-acryl (with weak fluorescence). Upon reaction with Cys, the acryloyl group on the phenolic oxygen atom of the Aza-acryl is removed, leading to the formation of Compound **1**, which exhibits strong fluorescence. Detection and imaging of Cys can be achieved by utilizing the changes in the fluorescence signal of the system. 

### 2.2. Spectra and Fluorescence Response

**Figure 2:**
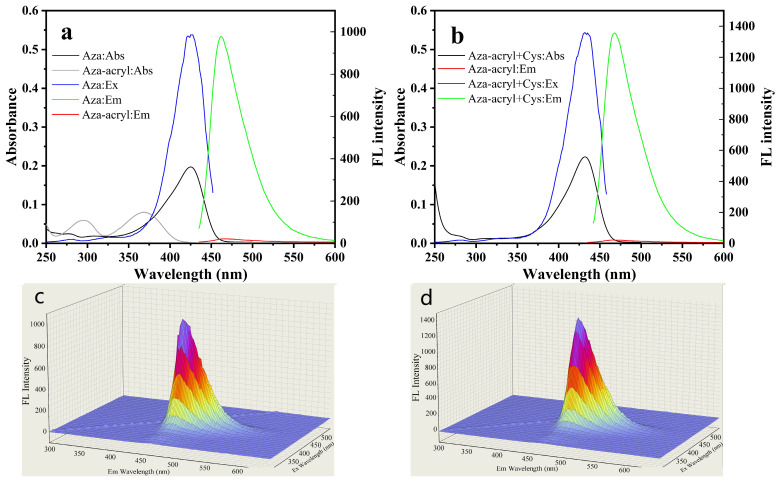
(**a**) UV-Vis absorption, fluorescence excitation/emission spectra of Aza (5 µM) and Aza-acryl (5 µM) in EtOH/phosphate buffer (20 mM, pH 7.4; 1:1 *v*/*v*); (**b**) UV-Vis absorption, fluorescence excitation/emission spectra of Aza-acryl (5 µM) + Cys (10 µM) in EtOH/phosphate buffer (20 mM, pH 7.4; 1:1 *v*/*v*); (**c**) fluorescence excitation–emission three-dimensional spectrum of Aza; (**d**) fluorescence excitation–emission three-dimensional spectrum of Aza-acryl + Cys. All experiments were conducted at room temperature.

Figure 2a shows the UV-Vis absorption and fluorescence spectra of Aza and Aza-acryl. The absorption peak of Aza is centered at 425 nm. Upon attachment of the acryl group, there are significant changes in the absorption spectrum of Aza-acryl. The absorption peak at 425 nm disappears, while two absorption peaks appear at 269 nm and 369 nm. This suggests significant changes in the distribution of electron cloud and energy levels of orbitals in the molecular structure, indicating a potentially significant electron transfer. Density functional theory (DFT) is employed to demonstrate the proposed proposition. Figure S1 displays the calculated UV-Vis spectra of Aza and Aza-acryl. The absorption peaks of Aza at 425 nm and Aza-acryl at 269 nm and 369 nm correspond to electron transitions from the orange orbitals to the green orbitals, respectively. These results highlight notable disparities in the molecular orbitals and electron distributions between Aza and Aza-acryl. Aza has maximum excitation and emission at 427 nm and 462 nm, respectively. The shape of Aza’s excitation spectrum is similar to its absorption spectrum, indicating that the fluorescence emission peak at 462 nm is derived from the absorption at 425 nm. Compared to Aza, the fluorescence emission intensity of Aza-acryl almost disappeared under excitation at 427 nm. The absolute quantum yields (QYs) of Aza and Aza-acryl were measured to be 49.75% and 1.15% (Figure S2), respectively, indicating that the fluorescence of Aza is significantly quenched by acryl groups.

Figure 2b illustrates the UV-Vis absorption and fluorescence spectra of Aza-acryl after reacting with L-Cys. It can be observed that the absorption peak at 432 nm was recovered, and the fluorescence emission (468 nm) intensity increases by a factor of 64 compared to Aza-acryl. Furthermore, Aza-acryl exhibits a consistent fluorescence response to equimolar concentrations of both L-Cys and D-Cys (Figure S3). Compound **1**, resulting from the reaction between Aza-acryl and Cys, was successfully isolated. The absolute QY of Compound **1** is 82.43%, indicating a significant enhancement in fluorescence emission. Compound **1** and Aza exhibit similar structures; however, Compound **1** demonstrates a significantly higher quantum yield compared to Aza. This can be attributed to the presence of an additional six-membered ring in Compound **1**, enhancing the molecular rigidity and minimizing non-radiative transitions. This suggests that Aza-acryl has the potential to be used as a fluorescent probe for detecting Cys. 

Figure 2c,d displays the three-dimensional excitation–emission spectrum of Aza and Aza-acryl + Cys. Both of the emission peak positions are independent of the excitation wavelength, representing typical molecular-state fluorescence emission behavior. 

### 2.3. Feasibility, Selectivity, and Sensitivity of Aza-acryl to Cys

**Figure 3:**
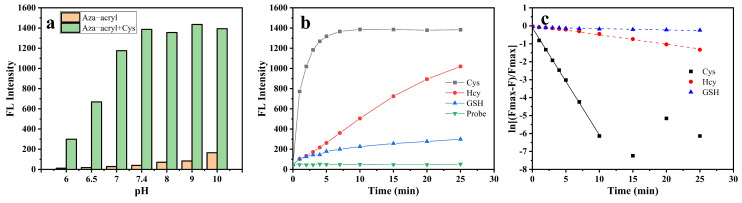
(**a**) pH-dependent fluorescence intensity of Aza-acryl (5 μM) without and with Cys (10 μM) in EtOH/phosphate buffer (20 mM, pH 7.4; 1:1 *v*/*v*); (**b**) time-dependent fluorescence intensity and (**c**) the pseudo-first-order rate constants of Aza-acryl (5 μM) with Cys/Hcy/GSH (10 μM) in EtOH/phosphate buffer (20 mM, pH 7.4; 1:1 *v*/*v*). λex/em = 432/468 nm. All experiments were conducted at room temperature.

In order to optimize the detection performance of Aza-acryl for Cys, the effects of pH and incubation time on the detection were measured. The influence of the pH values (6.0, 6.5, 7.0, 7.4, 8.0, 9.0, 10.0) on the fluorescence intensity of Aza-acryl and Aza-acryl + Cys system was studied, as shown in Figure 3a. As the pH value increased, the fluorescence intensity of the Aza-acryl solution increased at 468 nm. This could be due to alkalinity promoting the ester bond hydrolysis of the Aza-acryl, as well as the higher QY of the Aza fluorophore [35] under alkaline conditions. After the addition of Cys, the fluorescence intensity of the Aza-acryl solution significantly increases at various pH values. Within the pH range of 6–7.4, the fluorescence intensity of the Aza-acryl solution notably increases with pH. This is because, as the pH increases, the thiol group of Cys gradually ionizes and demonstrates stronger nucleophilicity, accelerating the addition reaction between the thiol group and Aza-acryl. Ultimately, pH 7.4 was selected as the optimal condition because the probe solution is relatively stable and demonstrates a significant response to Cys. Additionally, pH 7.4 aligns with physiological conditions.

In the presence of Cys, the fluorescence intensity of the Aza-acryl solution gradually increased with the pH value. Figure 3a also demonstrates that Aza-acryl as a probe has a significant response to Cys under physiological conditions (pH 7.4), indicating the feasibility of detecting Cys using this probe in biological systems.

Incubation time is an important parameter for probe performance, so the fluorescence intensity of Aza-acryl was evaluated as a function of incubation time in the presence of Cys/Hcy/GSH (Figure 3b). After adding Cys to the Aza-acryl solution, the fluorescence intensity of the system rapidly increased and reached equilibrium within 7.5 min. However, during this time range, the addition of Hcy/GSH resulted in a slow and slight increase in the fluorescence intensity. These results suggest that the reaction between Aza-acryl and Cys is rapid, while the reaction with Hcy/GSH is slow. This is determined by the differences in molecular structure between Cys, Hcy, and GSH. A detailed discussion of this aspect is presented in the subsequent section on reaction mechanisms.

Figure 3c shows the reaction kinetics curves of Aza-acryl with Cys, Hcy, and GSH based on Figure 3b. The apparent rate constants (k’) of the reactions were obtained using Equation (S1), and were 0.5973, 0.05218, and 0.0074 min^−1^ for Cys, Hcy, and GSH, respectively. The pseudo-first-order rate constant (k) of Cys was calculated as 1991 M^−1^ s^−1^, while those of Hcy and GSH were only 173.9 and 24.7 M^−1^ s^−1^, respectively. The reaction rate of Aza-acryl with Cys was 11.4 times that of Hcy and 80.6 times that of GSH, indicating the ability of Aza-acryl to effectively distinguish between Cys and Hcy/GSH within a few minutes.

Furthermore, the stability of Aza-acryl was evaluated. The stock solution of Aza-acryl (500 µM in DMSO) is stable. After 2 months of storage at room temperature, it has not changed and can still be used for precise analysis of Cys. However, when the stock solution is diluted to prepare the detection solution (by diluting 20 µL of the stock solution with EtOH/PBS = 1/1, 20 mM, pH 7.4), the fluorescence intensity of the detection solution slightly increases with time (Figure S4). Therefore, the stock solution of the probe can be stored for a long period, but once it is diluted to prepare the detection solution, it should be used promptly.

**Figure 4:**
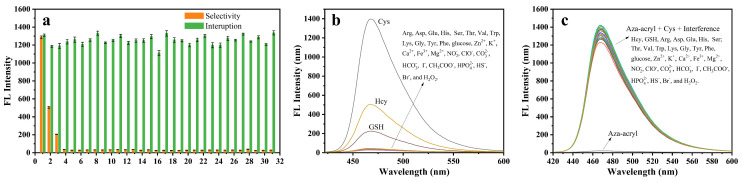
(**a**) Selectivity (orange) and sensitivity (green) responses of Aza-acryl (5 μM) toward Cys (10 μM) and different analytes (10 μM) in EtOH/phosphate buffer (20 mM, pH 7.4; 1:1 *v*/*v*). Error bars represent ±SD of three experiments. (**b**) The fluorescent recognition of Aza-acryl (5 μM) to Cys (10 μM) and other various species (10 μM) in EtOH/phosphate buffer (20 mM, pH 7.4; 1:1 *v*/*v*). (**c**) The fluorescent competitiveness of Aza-acryl (5 μM) after Cys (10 μM) is added in the presence of various other species (10 μM) in EtOH/phosphate buffer (20 mM, pH 7.4; 1:1 *v*/*v*). λex/em = 432/468 nm. All experiments were conducted at room temperature.

To investigate the specificity of Aza-acryl in detecting Cys, we examined its reactivity with various amino acids and ions. From Figure 4a,b, it is evident that Cys significantly enhances the fluorescence intensity of the probe, indicating a high selectivity of Aza-acryl towards Cys. Although 50 μM Hcy slightly increases the fluorescence intensity, considering its lower quantities in biological systems and the fact that the increase in fluorescence is limited, the probe is still able to effectively detect Cys. In order to evaluate the sensitivity of Aza-acryl in Cys detection, we also studied the potential interference of coexisting biomolecules under optimal experimental conditions, as shown in Figure 4a,c. The results indicate that the coexisting substances pose no apparent interfering effects on the detection of Cys.

### 2.4. Cys Titration Experiment

**Figure 5:**
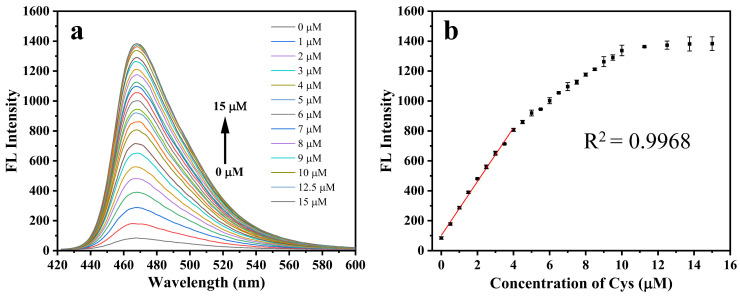
(**a**) Fluorescence spectra of Aza-acryl (5 μM) after 10 min in the presence of increasing concentrations of Cys in EtOH/phosphate buffer (20 mM, pH 7.4; 1:1 *v*/*v*); (**b**) relationship of fluorescence intensities and the concentrations of Cys (0–15 μM). Red line: Linear relationship of the fluorescence intensity to Cys (0.5–4 μM). Error bars represent ±SD of three experiments. λex/em = 432/468 nm. All experiments were conducted at room temperature.

Figure 5a,b depict the fluorescence recovery of Aza-acryl upon reaction with different concentrations of Cys. The result shows that the fluorescence intensity of the system increases progressively as the concentration of Cys rises. The fluorescence intensity of the system shows good linearity when the Cys concentration is 0.5–4 µM (R^2^ = 0.9968). The limit of detection (LOD) is determined to be 80 nM. Compared with some of the previously reported fluorescent probes for detecting Cys (Table S1), our probe has a similar detection limit and linear range. 

The fluorescence response in the range of Cys concentration from 0 to 10 µM exhibits nonlinearity, as depicted in Figure 5b. Two inflection points are observed at approximately 5 µM and 10 µM. Within the Cys concentration range of 0–5 µM, the ratio of Cys to Aza-acryl ranges from 0 to 1. The reaction between Cys and Aza-acryl may occur rapidly, resulting in a steep slope of the curve. However, despite the theoretical reaction ratio between Cys and Aza-acryl being 1/1, complete reaction of Aza-acryl (5 µM) is not achieved at a Cys concentration of 5 µM. Furthermore, in the Cys concentration range of 5–10 µM, the reaction rate between Cys and Aza-acryl may exhibit a decline, resulting in a gradual reduction in the slope of the curve. At a Cys concentration of 10 µM, the ratio of Cys/Aza-acryl corresponds to 2/1. At this stage, Aza-acryl is fully reacted, resulting in a stable fluorescence intensity that corresponds to a slope curve close to 0. Furthermore, the nonlinearity of the curve may also arise from the fluorescence inner-filter effect resulting from the overlap between the UV absorption of Aza-acryl and the excitation wavelength around 427 nm.

### 2.5. The Reaction Mechanism of Aza-acryl with the Thiols

The reaction mechanism of fluorescent probes based on acrylic acid phenyl esters for detecting biological thiols has been extensively studied [26,27,28,29,30,31,32,33,54,55,56,57,58,59,60]. The reaction involves two steps: first, the Michael addition of thiols to the probe’s double bond leads to the formation of linear thioethers. Second, the amino group of the biological thiol acts as a nucleophile, attacking the carbonyl group of the ester, resulting in the generation of cyclic thioethers and phenolic fluorescent substances. The structure of Compound **1** was confirmed by NMR, high-resolution mass spectra (HRMS), and IR. In this study, HPLC, ^1^H NMR, and HRMS were used to investigate the mechanism. The HPLC analysis results are presented in Figure S5, revealing the peak positions of 6.33 min and 4.21 min for the probe and Compound **1**, respectively. Upon mixing the probe with Cys, it is observed that the probe gradually transforms into Compound **1** during the reaction.

**Figure 6:**
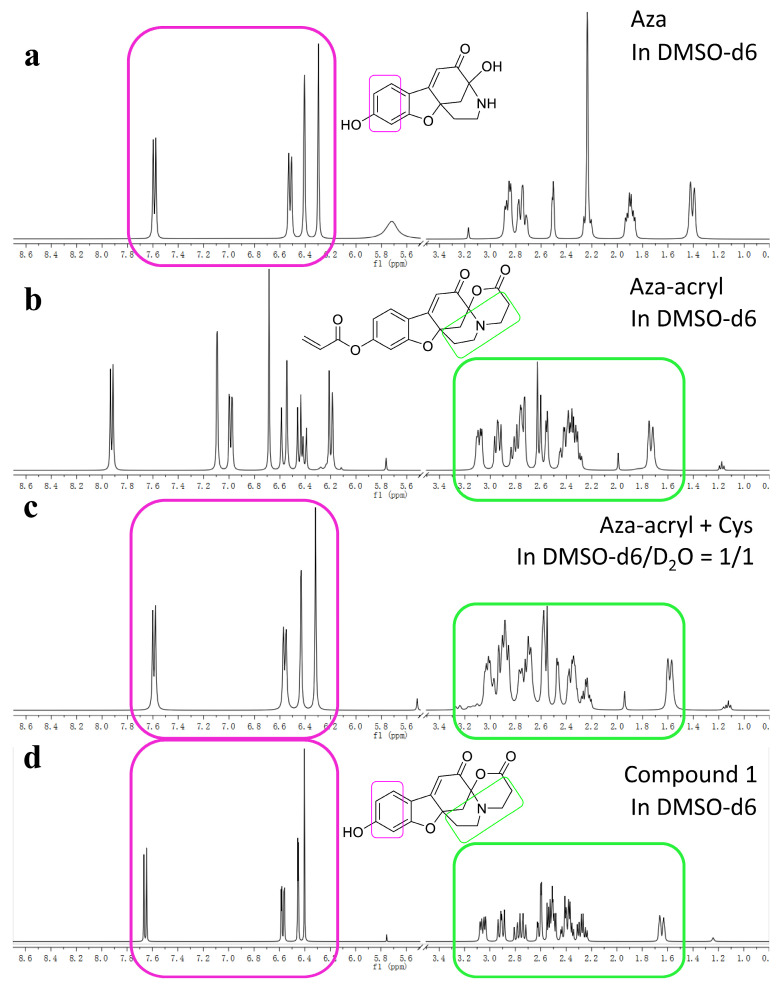
(**a**) The ^1^H NMR spectrum of Aza (10 mg/mL) in DMSO-d6; (**b**) the ^1^H NMR spectrum of Aza-acryl (10 mg/mL) in DMSO-d6; (**c**) the in situ ^1^H NMR spectrum of the reaction product of Aza-acryl (10 mg/mL) with Cys (5 mg/mL) and NaHCO_3_ (0.5 mg/mL) in D_2_O/DMSO-d6 = 1/1; (**d**) the ^1^H NMR spectrum of Compound **1** (10 mg/mL) in DMSO-d6. The peaks of DMSO and H_2_O, and the range of 3.5–5.5, were removed for clarity and compactness. All experiments were conducted at room temperature.

Figure 6a–d present the ^1^H NMR spectra of Aza, Aza-acryl, the reaction product of Aza-acryl with Cys, and Compound **1**, respectively. In Figure 6a, the spectrum region enclosed by a pink circle corresponds to the phenolic hydrogens in the molecular structure of Aza. Similarly, in Figure 6b, the spectrum region enclosed by a green circle corresponds to the aliphatic hydrogens of Aza-acryl. Figure 6c shows that, upon reaction with Cys, the aliphatic moiety of the product remains unchanged, while the aromatic ring moiety becomes identical to Aza. By comparison, it can be observed that the ^1^H NMR of Compound **1** (Figure 6d) is consistent with the product obtained from the reaction of Aza-acryl and Cys. The results indicate that, upon reaction with Cys, only the acryloyl group separates from Aza-acryl, while Aza-acryl undergoes a transformation into Compound **1**. 

Figures S17–S19 show the HRMS of Aza-acryl, Aza-acry+Cys, and Compound **1**, respectively. By comparing the molecular weights, it can be concluded that, upon reacting with Cys, Aza-acryl ([M+H]^+^ = 368.1135) undergoes the loss of a structural entity corresponding to an acryloyl group, and the MW of the product ([M+H]^+^ = 314.1031) is consistent with that of Compound **1** ([M+H]^+^ = 314.1033 or [M+Na]^+^ = 336.0850). Based on these results and previous research [31,33,54,55], it can be confirmed that the reaction product of the probe Aza-acryl with Cys is Compound **1**. Additionally, negative ion electrospray ionization MS analysis was conducted on the reaction products of Aza-acryl with Cys, Hcy, and GSH. This analysis revealed the presence of three mass spectrometry peaks: *m*/*z* = 174.0217, 188.0385, and 360.0861 respectively (Figures S20–S22), which can be attributed to three types of cyclic thioether byproducts. These findings further confirm the proposed mechanism.

Based on the MS, NMR, and kinetics results, and the literature [54,61], the possible detection mechanism is illustrated in Figure S23. Firstly, the thiol group of biological thiols possesses strong nucleophilicity under weak alkaline conditions at pH = 7.4, allowing it to undergo Michael addition with the double bond on the acryl group of Aza-acryl. Secondly, the amino group in biological thiols undergoes a nucleophilic reaction with carbonyls, resulting in the departure of the acryl group. Due to the proximity of the amino group in Cys to the carbonyl group and the formation of a stable seven-membered ring as the leaving group, the reaction occurs at a faster rate. On the other hand, the amino groups in Hcy and GSH are further from the carbonyl group and the resulting leaving groups are eight-membered and polyheterocyclic rings, resulting in slower reaction rates. Ultimately, another highly fluorescent product, Compound **1**, is formed, leading to the recovery of fluorescence and enabling the selective detection of biological thiols, especially Cys. 

**Figure 7:**
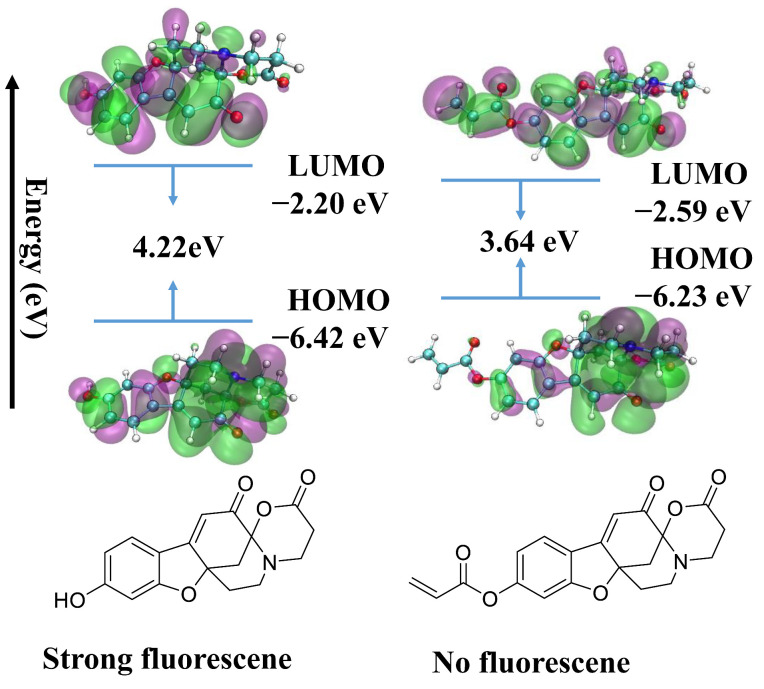
DFT optimized structures and frontier molecular orbitals (MOs) of Aza-acryl (**Left**) and Aza (**Right**).

In order to gain a better understanding of the sensing mechanism of Aza-acryl, DFT calculations were performed. The ground-state structures of Compound **1** and Aza-acryl were optimized using the ORCA 5.0.1 program, and the energy levels of their frontier molecular orbitals were calculated. As shown in Figure 7, the HOMO and LUMO of Compound **1** are mainly localized on the benzofuran moiety. The π electrons in the HOMO of Aza-acryl are primarily distributed on the benzofuran moiety, while a portion of the π electrons in the LUMO of Aza-acryl is transferred to the acryl group. The energy levels of the LUMO and HOMO support the possible photoinduced electron transfer (PET) process in Aza-acryl. The transfer of electrons from the benzofuran moiety (PET donor) to the acryl group (PET acceptor) weakens the fluorescence of the original fluorophore, corresponding to the fluorescence “off” state. Upon reaction with Cys, the acryl group is eliminated, and the PET process in the compound disappears, leading to fluorescence “on”. The energy gaps (HOMO–LUMO) between Aza-acryl and Compound **1** are calculated to be 3.64 and 4.22 eV, respectively, and this theoretical calculation matches the experimental results, rationalizing the PET process.

### 2.6. Imaging of Cys in Live Cells

Considering the excellent fluorescence response of Aza-acryl towards Cys, we investigated the potential application of Aza-acryl probe in live cell imaging. Firstly, the toxicity of Aza-acryl was examined. A549 cells were used for CCK-8 assay (Figure S24). After incubating the cells with different concentrations of Aza-acryl (0, 5, 10, 15, 20, 30, 50 uM) for 24 h, the cell viability remained consistently high, even at the highest concentration of 50 µM (over 89%). During cell imaging, the probe concentration was 5 µM, and the cell viability approached 100%. These results indicate the low toxicity of Aza-acryl towards cells and its good biocompatibility, establishing the prerequisites for its application in cellular experiments.

**Figure 8:**
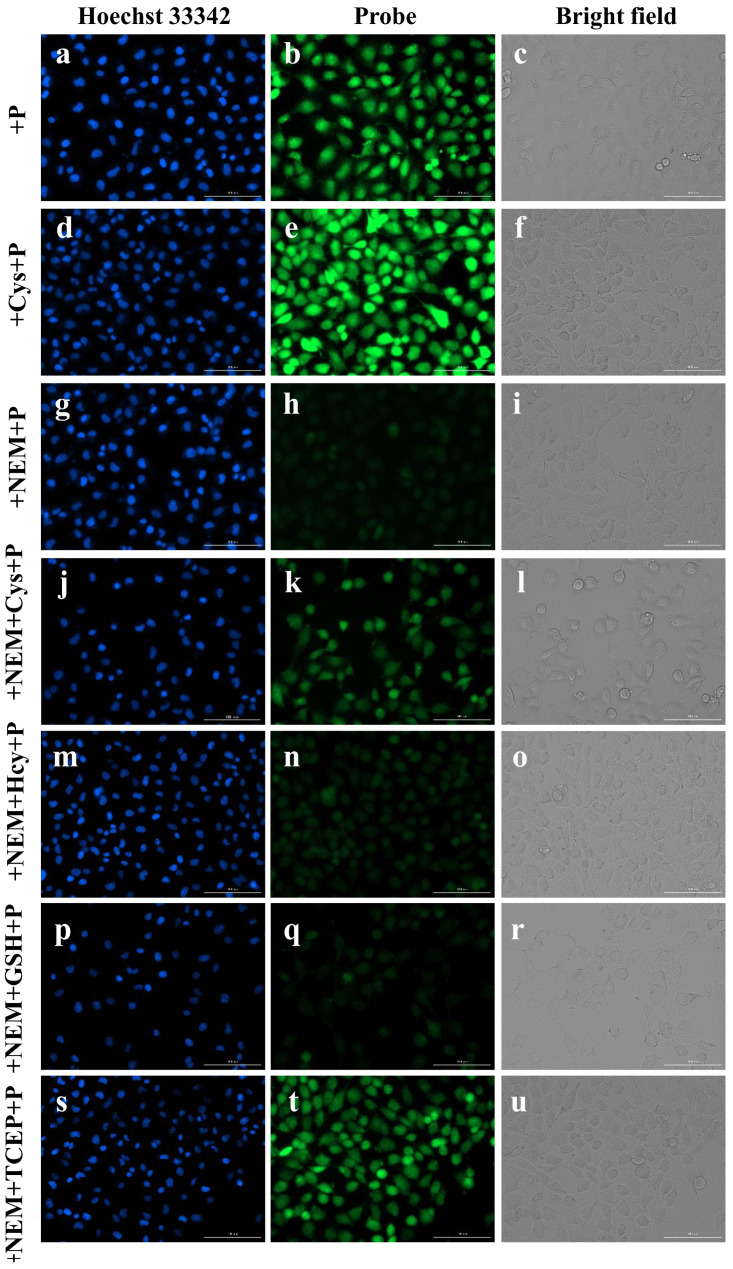
The fluorescence images of A549 cells. Hoechest 33,342 channel (λex = 377 nm and λem = 477 nm, column 1), Green channel (λex = 469 nm and λem = 525 nm, column 2), Bright field (column 3) in the presences of probe (**a**–**c**), Cys + probe (**d**–**f**), NEM + probe (**g**–**i**), NEM + probe + Cys (**j**–**l**), NEM + probe + Hcy (**m**–**o**), NEM + probe + GSH (**p**–**r**), and NEM + probe + TCEP (**s**–**u**). Scale bar: 100 μm.

Subsequently, the ability of Aza-acryl to image Cys in cells was investigated. After incubating the cells with the probe for 10 min, a homogeneous distribution of green fluorescence was observed throughout the entire cytoplasm or the surface of the cell (Figure 8a–c). Pre-treating the cells with exogenous Cys before incubation with the probe resulted in a more pronounced fluorescence signal (Figure 8d–f) compared to the previous group. Conversely, pre-treating the cells with N-ethylmaleimide (NEM), a thiol-blocking agent, to remove endogenous Cys, resulted in almost complete disappearance of the fluorescent signal in the cells (Figure 8g–i). The fluorescence intensities of cells are shown in Figure S25. These results indicate a notable correlation between the intracellular fluorescence and the cellular biothiol content. 

As shown in Figure 8j–l, the use of NEM to deplete all the endogenous biothiols was followed by the addition of exogenous Cys, which resulted in fluorescence recovery. However, the addition of exogenous Hcy (Figure 8m–o) and GSH (Figure 8p–r) did not lead to fluorescence recovery. This further demonstrates the high selectivity of Aza-acryl for Cys and its potential for imaging Cys in cells.

In biological systems, cysteine can exist not only in its free reduced form but also in an oxidized form with disulfide bonds. Tris(2-carboxyethyl)phosphine (TCEP) is a reducing agent that can break disulfide bonds and restore biothiols to their reduced form. The Aza-acryl was utilized to demonstrate this process. As shown in Figure 8s–u, cells were first treated with NEM to deplete all the endogenous biothiols, followed by a 30 min treatment with TCEP. The green fluorescence was observed in this group, indicating a significant conversion of Cys from its oxidized state to its reduced state. The result shown in Figure 8 supports the practical application of Aza-acryl for imaging Cys in cells.

### 2.7. Imaging of Cys in Zebrafish

**Figure 9:**
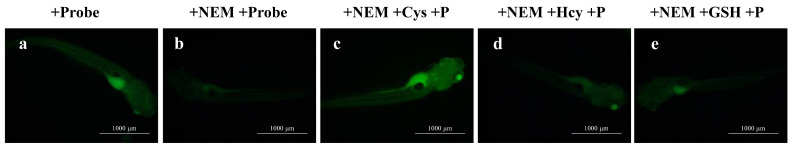
The fluorescence images of zebrafish larvae: (**a**) zebrafish incubated with Aza-acryl; (**b**) zebrafish pretreated by NEM, followed by incubation with Aza-acryl; (**c**–**e**) zebrafish pretreated with NEM, followed by treatment with Cys, Hcy, or GSH before being incubated with Aza-acryl. Scale bar: 1000 μm.

Encouraged by results of the probe to detect Cys in cells, we subsequently employed zebrafish as an animal model to investigate the imaging capability of the probe for Cys in vivo. Zebrafish is often used as an animal experiment model because of its transparent body in early development, and due to its rapid development, easy reproduction and maintenance, genetic operability, and high conservation with the human genome. After co-incubation of Aza-acryl with 6-day-old zebrafish, bright green fluorescence was observed (Figure 9a). Pre-treatment of zebrafish with NEM, which depletes endogenous Cys, followed by co-incubation with Aza-acryl, resulted in a significant decrease in the fluorescent signal (Figure 9b). Subsequent addition of exogenous Cys after depleting biothiols led to a substantial recovery of green fluorescence (Figure 9c). However, the addition of exogenous Hcy (Figure 9d) and GSH (Figure 9e) did not result in a significant recovery of fluorescence. The fluorescence intensities of zebrafish are shown in Figure S26. These results demonstrate the ability of the Aza-acryl probe to image Cys in live zebrafish.

## 3. Materials and Methods

### 3.1. Materials

Resorcinol, dopamine hydrochloride, triethylamine, acryloyl chloride, cysteine, homocysteine, glutathione, and other amino acids were obtained from Aladdin Biochemical Technology Co., Ltd., Shanghai, China. Various inorganic salts were obtained from Guangfu Technology Development Co., Ltd., Tianjin, China. Organic solvents were purchased from Yongda Chemical Reagent Co., Ltd., Tianjin, China and re-distilled before use. 

### 3.2. Instruments

NMR spectra were measured using a technologies plus-400 Mr instrument (Agilent Company, Lexington, MA, USA). The mass spectra were measured using a Water G2-XS Q-TOF instrument (Waters, Milford, MA, USA). The HPLC experiment was conducted using a LC-15C instrument (Shimadzu, Nakagyo-ku, Japan). The absolute quantum yield (QY) was obtained using a Horiba fluoromax-4 spectrometer and the integrating sphere method. Fluorescence spectra were obtained using a Cary Eclipse fluorescence spectrometer (Aligent Company, Lexington, MA, USA). The single crystal structure was obtained using a Bruker smart Apex X-ray single crystal diffractometer (Bruker, Billerica, MA, USA). UV-Vis absorption spectra were obtained by a Cary 50 (Varian Company, Palo Alto, CA, USA). The zebrafish fluorescence images were captured using an inversed fluorescent microscope (MSHOT MF53). The cellular and zebrafish fluorescence images were captured using a Cytation 5 Cell Imaging Multi-Mode Reader (Aligent Company, USA). 

### 3.3. Synthesis

#### 3.3.1. Synthesis of 4,9-Dihydroxy-1,2,3,4-tetrahydro-5H-4,11a-methanobenzofuro[2,3-d]azocin-5-one (Aza)


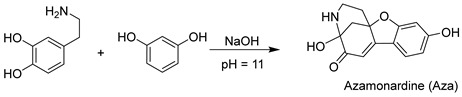


Aza was synthesized according to the literature method with some modifications [35,44]. Resorcinol (440.44 mg, 4.00 mmol) and dopamine hydrochloride (758.56 mg, 4.00 mmol) were added to a 500 mL beaker, followed by the addition of 250 mL of ultrapure water and stirring until the solids had dissolved. The pH was adjusted to 11, and the mixture was stirred vigorously at room temperature and under ambient conditions for 24 h. Upon completion of the reaction, the pH was lowered to neutral, and the mixture was extracted with ethyl acetate. The organic layer was subsequently washed with ultrapure water three times, dried, and the solvent was removed to yield a bright yellow Aza product (128 mg, 10.7% yield). ^1^H NMR (400 MHz, DMSO-*d*_6_) δ 10.52 (s, 1H), 7.58 (d, *J* = 8.5 Hz, 1H), 6.51 (dd, *J* = 8.4, 2.1 Hz, 1H), 6.40 (d, *J* = 2.0 Hz, 1H), 6.29 (s, 1H), 5.71 (s, 1H), 2.85 (dd, *J* = 13.0, 5.6 Hz, 1H), 2.75 (dt, *J* = 16.6, 8.3 Hz, 1H), 2.23 (s, 2H), 1.89 (td, *J* = 12.5, 5.8 Hz, 1H), 1.56–1.28 (m, 1H). ^13^C NMR (101 MHz, DMSO-*d*_6_) δ 196.47, 165.54, 164.48, 163.00, 125.97, 112.92, 112.04, 111.12, 97.86, 89.41, 82.74, 46.74, 36.96, 31.86. HRMS: *m*/*z* [C_14_H_14_NO_4_]^+^ calcd 260.0917, found 260.0923. 

#### 3.3.2. Synthesis of 2,14-Dioxo-3,4,6,7-tetrahydro-2H,14H-7a,14a-methanobenzofuro[3,2-e][1,3]oxazino[3,2-a]azocin-10-ylacrylate (Aza-acryl)


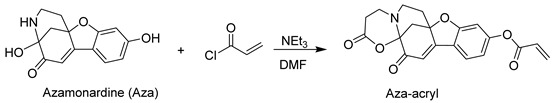


Aza (100.00 mg, 0.39 mmol) was placed in a dried reaction vial sealed with a rubber septum and the reaction setup was placed in a fume hood. Two milliliters of anhydrous DMF were injected into the system, and the Aza was stirred until fully dissolved. The reaction vial was then placed in an ice bath, and 200 μL (1.43 mmol) of anhydrous triethylamine and 100 μL (1.23 mmol) of acryloyl chloride were added and stirred. After 6 h of reaction at room temperature, DMF was removed under vacuum using a vacuum pump, and the crude product was purified by flash column chromatography (DCM/ethyl acetate = 25:1) to yield a bright yellow Aza-acryl (92 mg, 65.2% yield). ^1^H NMR (400 MHz, DMSO-*d*_6_) δ 7.93 (d, *J* = 8.4 Hz, 1H), 7.10 (d, *J* = 2.0 Hz, 1H), 6.98 (dd, *J* = 8.4, 2.0 Hz, 1H), 6.68 (s, 1H), 6.63–6.49 (m, 1H), 6.42 (dd, *J* = 17.2, 10.3 Hz, 1H), 6.19 (d, *J* = 10.0 Hz, 1H), 3.08 (dd, *J* = 11.9, 5.0 Hz, 1H), 2.93 (dd, *J* = 11.7, 8.4 Hz, 1H), 2.86–2.69 (m, 2H), 2.61 (d, *J* = 10.6 Hz, 1H), 2.55 (d, *J* = 5.1 Hz, 1H), 2.46–2.24 (m, 3H), 1.81–1.65 (m, 1H). ^13^C NMR (101 MHz, DMSO-*d*_6_) δ 188.65, 167.41, 164.19, 163.66, 162.58, 155.84, 134.34, 127.32, 125.97, 119.39, 116.73, 115.49, 106.11, 90.91, 88.20, 44.99, 44.55, 43.87, 31.72, 29.00. HRMS: *m*/*z* [C_20_H_18_NO_6_]^+^ calcd 368.1129, found 368.1135.

### 3.4. Crystal Structure Analysis

Crystal data for Aza-acryl: monoclinic, space group P2_1_/c (no. 14), *a* = 7.9996(4) Å, *b* = 28.3875(11) Å, *c* = 7.4750(3) Å, *β* = 93.604(2)°, *V* = 1694.13(13) Å^3^, *Z* = 4, *T* = 170.0 K, μ(MoKα) = 0.107 mm^−1^, *Dcalc* = 1.440 g/cm^3^, 14,302 reflections measured (5.102° ≤ 2Θ ≤ 52.712°), 3439 unique (*R*_int_ = 0.0620, R_sigma_ = 0.0511), which were used in all calculations. The final *R*_1_ was 0.0440 (I > 2σ(I)) and *wR*_2_ was 0.1101 (all data). Goodness-of-fit on F^2^ = 1.077. Crystallographic data have been deposited at the Cambridge Crystallographic Data Center as supplementary publications (CCDC 2283126).

### 3.5. General Spectral Analysis

The compound or probe stock solution was prepared in dimethyl sulfoxide (DMSO), followed by dilution to 5 μM using phosphate-buffer (20 mM, PBS) solution for absorbance or fluorescence spectroscopy measurements. After the addition of Cys or other substances in the reaction system (PBS, 37 °C), the absorbance or fluorescence spectra were measured using UV-Vis or fluorescence spectrometer. Each experiment was repeated three times. The procedures for pH, incubation time, selectivity, sensitivity, and titration experiments are described in detail in the supporting information.

### 3.6. Imaging

#### 3.6.1. Cell Imaging

The experiment was divided into seven groups. In the first group (probe group), cells were incubated with Aza-acryl (5 μM) for 10 min. Prior to imaging, cells were washed three times with PBS to remove any extracellular Aza-acryl. In the second group (Cys+probe group), cells were incubated with Cys (100 μM) for 20 min, followed by three washes with PBS and subsequent incubation with Aza-acryl (5 μM) for 10 min before imaging. In the third group (NEM+probe group), cells were pre-treated with N-ethylmaleimide (NEM, 100 μM) at 37 °C for 40 min. After that, cells were incubated with Aza-acryl (5 μM) for 10 min prior to imaging.

In the fourth to seventh groups (NEM+Probe+Cys/Hcy/GSH/TECP groups), cells in each group were first pre-treated with NEM (100 μM) for 40 min. After three washes with PBS, the cells were incubated with Cys, Hcy, GSH, or TECP (100 mM) at 37 °C for 20 min. Subsequently, cells in each group were incubated with Aza-acryl (5 μM) for an additional 10 min before imaging. All fluorescence images were captured using a Cytation 5 multi-mode plate reader. Hoechst 33,342 was used for live cell nuclear staining. The Hoechst 33,342 channel was collected at 477 nm, while the green channel was collected at 525 nm.

#### 3.6.2. Zebrafish Imaging

Zebrafish were cultured in a 12-well plate with 1 mL of nutrient solution added per well. Zebrafish were anesthetized with MS-222 prior to the experiment. In the control group, Aza-acryl (5 μM) was added and incubated for 5 min before fluorescence imaging. In the NEM group, NEM (100 μM) was added and incubated for 20 min, followed by incubation with Aza-acryl (5 μM) for 5 min before fluorescence imaging. In the NEM+Cys, NEM+Hcy, and NEM+GSH groups, NEM (100 μM) was first incubated for 20 min, followed by incubation with Cys, Hcy, and GSH solutions (100 μM) for 20 min. The nutrient solution was then replaced, followed by incubation with Aza-acryl (5 μM) for 5 min before fluorescence imaging.

## 4. Conclusions

In this study, we successfully designed and synthesized a novel biocompatible fluorescence probe, Aza-acryl, by utilizing azamonardine as the fluorophore. Structural characterization through X-ray crystallography, NMR, and mass spectrometry confirmed that Aza-acryl is a compound with a rigid five-membered ring skeleton. Computational analysis based on density functional theory further elucidated that the fluorescence of the Aza fluorophore was effectively quenched by the acryl group through the PET effect. After the reaction with Cys, the fluorescence intensity of the system increased by a factor of 64. Moreover, kinetic experiments revealed that Cys can rapidly eliminate the acryl group. The reaction rate of Aza-acryl with Cys was 11.4 times higher than that of Hcy and 80.7 times higher than that of GSH, enabling effective discrimination of Cys from other biological thiols (Hcy/GSH). The probe exhibited excellent selectivity towards Cys, with a low detection limit (80 nM), rapid response (10 min), and low cytotoxicity. Aza-acryl was successfully applied to fluorescence imaging of Cys in both cellular and zebrafish models. The novel Aza-acryl probe holds great potential as a powerful imaging tool for the research and monitoring of Cys in vitro and in vivo.

## Data Availability

Data are contained within the article or Supplementary Materials.

## Supplementary Materials

The following supporting information can be downloaded at: https://www.mdpi.com/article/10.3390/molecules28176246/s1. Procedures for general spectral analysis, the calculation method for LOD, kinetic studies, mechanistic experiment, cells culture, and CCK-8 assay; Figure S1: The calculated UV-Vis spectra of Aza and Aza-acryl via DFT; Figure S2: Quantum yield of (a) Aza (5 µM), (b) Aza-acryl (5 µM), and (c) Compound **1** (5 µM) in ethanol/PBS (pH = 7.4, 20 mM) =1/1 (*v*/*v*). All experiments were conducted at room temperature; Figure S3: The fluorescence response of Aza-acryl (5 µM) to L-Cys (4 µM) and D-Cys (4 µM); Figure S4: the stability of the probe (5 µM) in detection solution (EtOH/PBS = 1/1, 20 mM, pH 7.4, 25 °C); Figure S5: HPLC of Aza-acryl (5 µM in DMSO), Compound **1** (5 µM in DMSO), and the product of Aza-acryl (5 µM) + Cys (10 µM) in EtOH/phosphate buffer (20 mM, pH 7.4; 1:1 *v*/*v*) at 1 min, 2.3 min, 5 min, and 10 min. All experiments were conducted at room temperature (r.t.); Figure S6: ^1^H NMR spectra of Aza (10 mg/mL) in DMSO-d6 at r.t.; Figure S7: ^13^C NMR of Aza (10 mg/mL) in DMSO-d6 at r.t.; Figure S8: ^1^H NMR of Aza-acryl (10 mg/mL) in DMSO-d6 at r.t.; Figure S9: ^13^C NMR of Aza-acryl (10 mg/mL) in DMSO-d6 at r.t.; Figure S10: ^1^H NMR Spectra of the reaction product of Aza-acryl (10 mg/mL) with Cys (5 mg/mL) and NaHCO_3_ (0.5 mg/mL) in D_2_O/DMSO-d6 = 1/1 at r.t.; Figure S11: 1H NMR spectra of Compound **1** (10 mg/mL) in DMSO-d6 at r.t.; Figure S12: ^13^C NMR spectra of Compound **1** (10 mg/mL) in DMSO-d6 at r.t.; Figure S13. HSQC spectra of Compound **1** (5 mg/mL) in DMSO-d6 at r.t.; Figure S14. HMBC spectra of Compound **1** (5 mg/mL) in DMSO-d6 at r.t.; Figure S15. IR spectra of Compound **1**; Figure S16: Positive ion ESI mass spectra of the Aza; Figure S17: Positive ion ESI mass spectra of the Aza-Aryl; Figure S18: Positive ion ESI mass spectra of Compound **1**; Figure S19: Positive ion ESI mass spectra of Aza-Aryl+Cys; Figure S20: Negative ion ESI mass spectra of Aza-Aryl+Cys; Figure S21: Negative ion ESI mass spectra of Aza-Aryl+Hcy; Figure S22: Negative ion ESI mass spectra of Aza-Aryl+GSH; Figure S23 The possible reaction mechanism of Aza-acryl with three biological thiols; Figure S24: Cytotoxicity of 0–50 μM Aza-Aryl toward A549 cells by CCK-8 assay; Figure S25: Fluorescence intensities of A549 cells in Figure 8b–t; Figure S26: Fluorescence intensities of zebrafishes in Figure 9a–e; Table S1: Comparisons of linear range and limit of detection (LOD) of previously reported fluorescent probes for detecting Cys.

## Sample Availability

Resorcinol, dopamine hydrochloride, and acryloyl chloride are available from the authors.
