# Supplementary material for: A Novel Fluorescence Probe Based on Azamonardine for Detecting and Imaging Cysteine in Cells and Zebrafish with High Selectivity and Sensitivity
Source: Molecules. 2023 Aug 25;28(17):6246. doi: 10.3390/molecules28176246 (PMC10488781; doi:10.3390/molecules28176246)
Supplement: Supplementary file 1 [file molecules-28-06246-s001.zip › molecules-2547253-supplementary.pdf]

Supporting Information

A novel fluorescence probe based on Azamonardine for detecting and imaging Cysteine in Cells and Zebrafish with highly selectivity and sensitivity

Yixu Zhao [1,2], Ting Wang [3], Ahmed Mohammed Ali Abdulkhaleq [4], Zhongfu Zuo [1,2,*], Yongjin Peng [5,*], Xibin Zhou [4,*]

[1] Department of Anatomy, Histology and Embryology, Jinzhou Medical University, 121001, Jinzhou, P. R. China

[2] Liaoning Key Laboratory of Diabetic Cognitive and Perceptive Dysfunction, Jinzhou Medical University, 121001, Jinzhou, P. R. China

[3] Faculty of Life Sciences and Technology, Kunming University of Science and Technology, Kunming, P. R. China

[4] College of Pharmacy, Jinzhou Medical University, Jinzhou, P. R. China

[5] College of Basic Science, Jinzhou Medical University, Jinzhou, P. R. China

* Correspondence: zuozhongfu@jzmu.edu.cn (Z.F.Z); pyj@jzmu.edu.cn (Y.J.P); dawei492@jzmu.edu.cn (X.B.Z)

Experimental Section

1 General spectral analysis

1.1 pH to fluorescence responses of Aza-acryl probe.

Stock solutions of Aza-acryl at a concentration of 0.5 mmol/L and Cys at a concentration of 1 mmol/L were prepared in advance. A series of PBS solutions with concentrations of 20 mmol/L and pH values ranging from 6 to 10 were prepared, and then mixed with same volume ethanol to generate a series of buffer solutions for further use. For fluorescence spectroscopy analysis, 1.98 mL of each buffer solution at different pH values were taken and mixed with 20 μL of the 0.5 mmol/L Aza-acryl stock solution, followed by 10 minutes of incubation. For the next analysis, 1.96 mL of each buffer solution at different pH values were taken and mixed with 20 μL of the 0.5 mmol/L Aza-acryl stock solution and 20 μL of the 1 mmol/L Cys stock solution, followed by 10 minutes of incubation before fluorescence spectroscopy analysis.

1.2 Time to fluorescence responses of Aza-acryl probe

A stock solution of Aza-acryl with a concentration of 0.5 mmol/L was prepared. Stock solutions of Cys, Hcy and GSH with a concentration of 1 mmsol/L were respectively prepared. In the control group, 20 μL of 0.5 mmol/L Aza-acryl stock solution were added to 1.98 mL of buffer with pH 7.4. The fluorescence spectra of the mixture were recorded at different time. In the experimental group, 20 μL of 0.5 mmol/L Aza-acryl stock solution were added to three 1.96 mL portions of buffer with pH 7.4. Then, 20 μL of each 1 mmol/L stock solution of Cys, Hcy, and GSH were added to the corresponding solutions. The fluorescence spectra of these mixed solutions were recorded at different time points.

1.3 Cys titration experiment

Take 1 mL of the buffer solution mentioned above with a pH of 7.4 and add 20 μl of 0.5 mmol/L Aza-acryl mother liquor followed by a specific volume (0 -30μL) of 1 mmol/L Cys mother liquor. Adjust the total volume to 2 mL by adding buffer

solution. Mix well and let sit for 10 minutes, then measure its fluorescence spectrum.

1.4 The calculation method for LOD

The calculation formula for LOD is shown below:

$LOD = 3\sigma / k$

In this formula, σ represents the standard deviation of the fluorescence intensity of probe solution (Not add Cys), and k represents the slope of the calibration curve.

To calculate the LOD, first, a set of probe solution (5 μM) is prepared and measured. The fluorescence intensity at 468 nm of a set of probe solution (5 μM) obtained are used to determine the standard deviation (σ).

Next, a set of Cys solution (0.5-15 μM) is prepared. After react with the probe (5 μM), a calibration curve is constructed using the concentrations of the Cys and the corresponding fluorescence intensity (the experiment for each concentration was repeated three times). Endeavor to choose a linear range of variation. The slope of this calibration curve (k) is determined.

Finally, the LOD is calculated by dividing 3 times the standard deviation (3σ) by the slope of the calibration curve (k). The LOD represents the lowest concentration of the analyte that can be reliably detected above the background noise or baseline signals.

1.5 Selectivity and sensitivity of Aza-acryl to detect Cys

Prepare a stock solution of 1 mmol/L arginine, aspartic acid, glutamic acid, histidine, serine, threonine, leucine, tryptophan, lysine, glycine, tyrosine, phenylalanine, cysteine, homocysteine, glutathione, glucose, $ZnSO_4$, KI, $CaCl_2$, $FeCl_3$, $MgSO_4$, $NaNO_2$, NaClO, Na_2CO_3, $NaHCO_3$, CH_3COONa, $NaHPO_4$, NaHS, NaBr, and H_2O_2.

For the selective experiment, multiple 1 mL portions of the pH = 7.4 buffer were taken. Each solution was added 20 μL Aza-acryl stock solution. Then, 20 μL of each amino acid and ion stock solution was added to each solution. Adjust the total

volume to 2 mL by adding buffer solution. The fluorescence spectra of these mixed solutions were recorded after 10 minutes.

As for the interference experiment, multiple 2 mL portions of the aforementioned buffer solution with a pH of 7.4 were taken. Each solution was added 20 μL Aza-acryl stock solution. Then, 20 μL of each amino acid and ion stock solution were added to each solution, and the mixture was uniformly mixed. 20 μL of Cys stock solution was then added to each solution, and the fluorescence spectra were recorded after 10 minutes.

2. Kinetic studies.

The rate constant was determined from the fluorescence titration data based on a reported method. The reaction of the probe Aza-acryl (5 μM) with Cys in PBS (20 mM, pH 7.4) was monitored using the fluorescence intensity at 462 nm. The reaction was carried out at room temperature. By fitting the fluorescence intensity of the sample to the apparent first-order k′ Equation S1, the apparent first-order rate constant is determined:

$$\ln [(F_{max} - F_t)/F_{max}] = -k't \qquad \text{(S1)}$$

Where Ft is the fluorescence intensity at t and Fmax is the maximum fluorescence intensity when the reaction is complete. k′ is the apparent rate constant. The pseudo-first-order rate constant k ($M^{-1}\ s^{-1}$) was obtained from Equation S2,

$$k' = kC \qquad \text{(S2)}$$

where C is the concentration of Cys, Hcy, or GSH.

3 Mechanistic experiment

3.1 Isolation and Characterization of Compound 1

The Aza-acryl (40 mg, 0.11 mmol) was dissolved in 10 mL of DMSO. Under vigorous stirring, 5 mL of Cys (10.5 mg/mL) and 5 mL of $NaHCO_3$ (10 mg/mL) aqueous solution were added. The color of the solution changed from pale yellow to bright green. Stirring was continued for 30 minutes, and then 50 mL of dichloromethane was added. HCl aqueous solution (0.7 mol/L) was added under

stirring to adjust the pH to 6.5, at which point the solution became colorless. The CH_2Cl_2 layer was washed with water (50 mL × 3) and dried over anhydrous sodium sulfate. After removing the solvent using a rotary evaporator, a bright yellow solid was obtained. Impurities were removed by washing with ethanol (5 mL × 3), yielding compound 1 (yield of 42%). ^1H NMR (400 MHz, DMSO-d6) δ 10.71 (s, 1H), 7.66 (d, J = 8.5 Hz, 1H), 6.57 (dd, J = 8.5, 2.1 Hz, 1H), 6.45 (d, J = 2.0 Hz, 1H), 6.40 (s, 1H), 3.11 – 3.01 (m, 1H), 2.91 (dd, J = 11.6, 8.4 Hz, 1H), 2.76 (ddd, J = 18.3, 10.2, 8.4 Hz, 1H), 2.61 (dd, J = 10.5, 2.5 Hz, 1H), 2.56 – 2.46 (m, 2H), 2.46 – 2.33 (m, 2H), 2.27 (td, J = 12.6, 5.5 Hz, 1H), 1.64 (dq, J = 12.4, 2.3, 1.8 Hz, 1H). ^{13}C NMR (101 MHz, DMSO) δ 29.504743, 32.656830, 44.446872, 45.074925, 45.708217, 88.404685, 91.452228, 98.381420, 112.146604, 112.190236, 113.134915, 126.863754, 164.425893, 165.596413, 166.464114, 167.962250, 188.126089. HRMS: m/z $[C_{17}H_{15}NO_5+H]^+$ calcd 314.1023, found 314.1033; m/z $[C_{17}H_{15}NO_5+Na]^+$ calcd 336.0842, found 336.0850. IR (cm^{-1}) 3432, 3162, 2939, 2852, 1752, 1603, 1565, 1481, 1362, 1275, 1231, 1205, 1165, 1105, 1037.

3.2 MS experiment

Aza and Aza-acryl was directly analyed by MS. For the compound 1, 20 μL of Aza-acryl stock solution and 20 μL of Cys stock solution were added to 2 mL of H_2O and add 0.5 μL NH_3OH. The mixture was mixed uniformly and left to stand for 10 minutes before conducting HPLC-MS analysis (Waters BEH C_{18} (2.1×50 mm, 1.7 μm) column, with a 2 μL injection volume, a flow rate of 1 mL/min, and eluted in a gradient of CH_3OH/H_2O = 20/80-90/10).

3.3 NMR experiment

Aza and Aza-acryl was directly analyed by NMR. For the compound 2, 10 mg of pure Aza-acryl powder was dissolved in 0.5 mL of deuterated DMSO. 5 mg of dried Cys and 0.5 mg of dried $NaHCO_3$ were dissolved in 0.5 mL of deuterated water. Mix the above-mentioned solutions and left to stand for 30 minutes before centrifuging. The upper clear liquid was taken for ^1H NMR analysis.

3.4 HPLC experiment

Prepare separate DMSO stock solutions for Aza-acryl (500 μM) and compound 2 (500 μM). Also, prepare separate water stock solutions for Cys (1 mM). For Aza-acryl and compound 2, dilute 100 μL of Aza-acryl or compound 2 into 900 μL of DMSO and analyze using HPLC. For Aza-acryl+Cys, combine 100 μL of Aza-acryl stock solution with 800 μL of PBS/ethanol solvent, then add 100 μL of Cys stock solution. After thorough mixing, take product solutions at different reaction time points for HPLC analysis (using a SHISHIDO C_{18} column (4.6×250 mm, 5 μm) with a 10 μL injection volume, a flow rate of 1 mL/min, and elution using a CH_3OH/H_2O gradient of 63/37).

4 Cells culture and CCK-8 assay

The human lung cancer cell line A549 were treated in Roswell Park Memorial Institute 1640 medium (RPMI-1640) supplemented and seeded on 96-well plates which were placed in an incubator at 37 °C with 5% CO_2 for 24 h. The RPMI-1640 was appended with 10% fetal bovine serum (FBS), 100 $U·mL^{-1}$ penicillin, and 100 $μg·mL^{-1}$ streptomycin.

The CCK-8 assay was used to evaluate the cytotoxicity of Aza-acryl on A549 cells. After removing the culture medium, the fresh culture medium and probes of different concentrations were added to the cells in sequence and continued to incubate for 24 h at 37℃. Subsequently, 10 μL of CCK-8 was added to the cells and incubated for 2.0 h. The absorbance value of each well was measured at OD 450 nm of enzyme-linked immunosorbent assay instrument. Five experiments were performed for each individual concentration and cell viability was assessed using the mean ± standard deviation of the data.

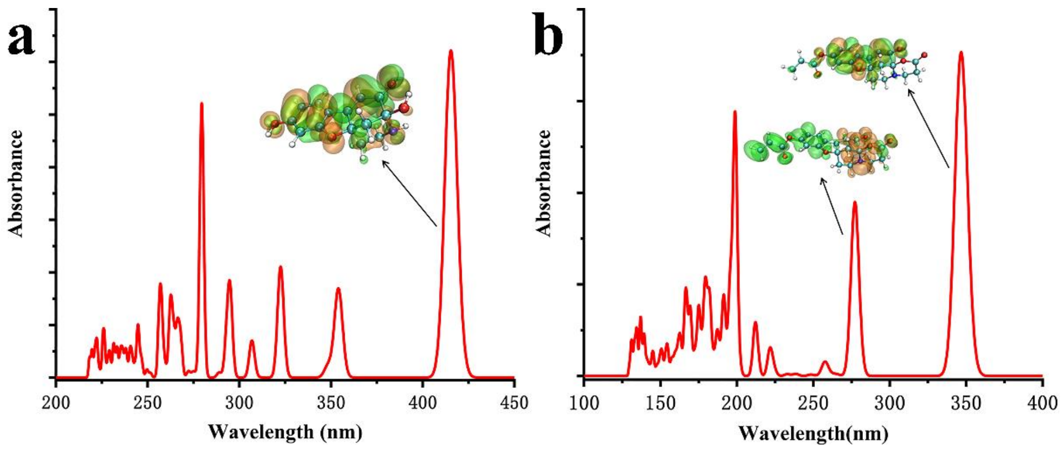

Figure S1. The calculated UV-Vis spectra of Aza and Aza-acryl by DFT.

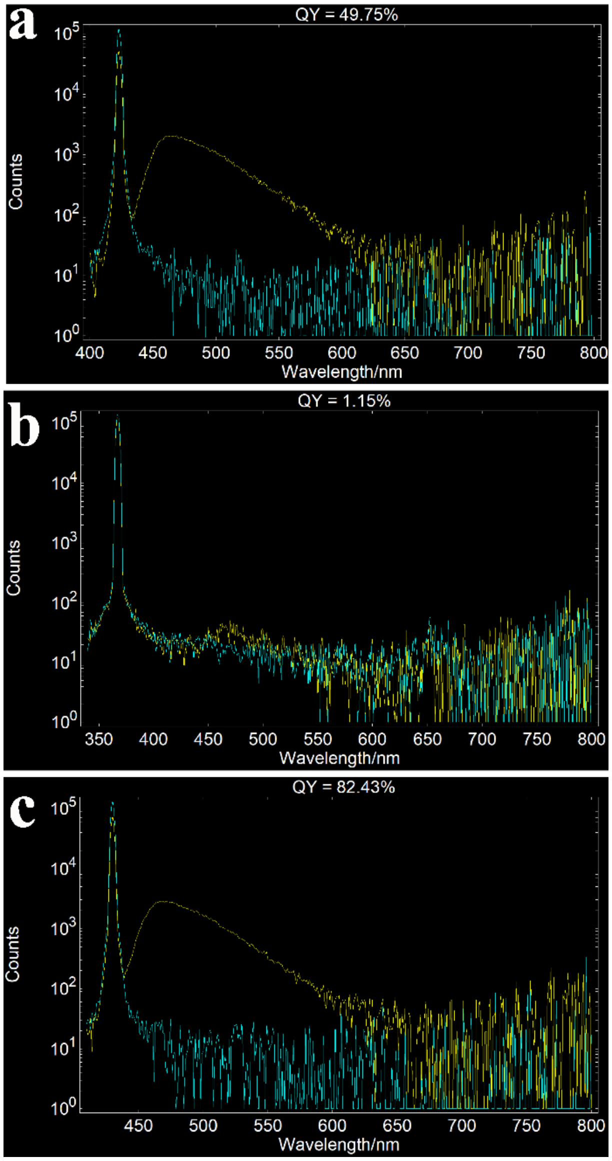

Figure S2. Quantum yield of (a) Aza (5 μM), (b) Aza-acryl (5 μM), and (c) Compound 1 (5 μM) in ethanol/ PBS (pH=7.4, 20mM) =1/1 (v/v). All experiments were conducted at room temperature.

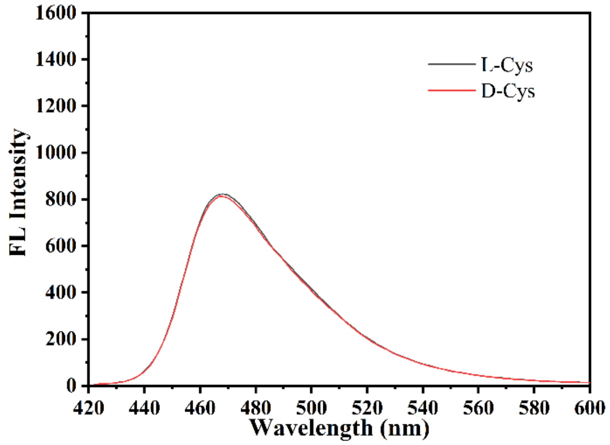

Figure S3. The fluorescence response of Aza-acryl (5 μM) to L-Cys (4 μM) and D-Cys (4 μM).

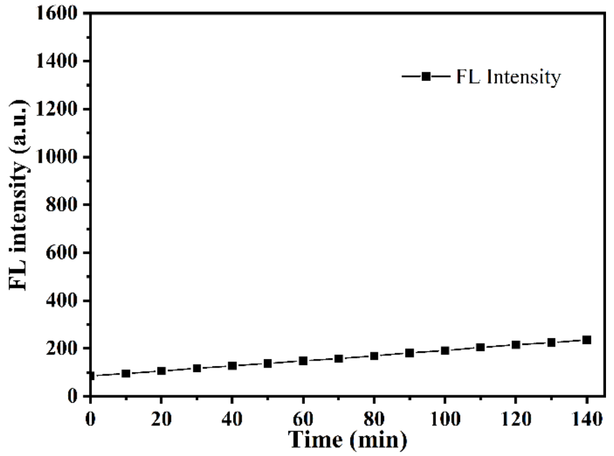

Figure S4. the stability of the probe (5 μM) in detection solution (EtOH/PBS = 1/1, 20 mM, pH 7.4, 25 ℃)

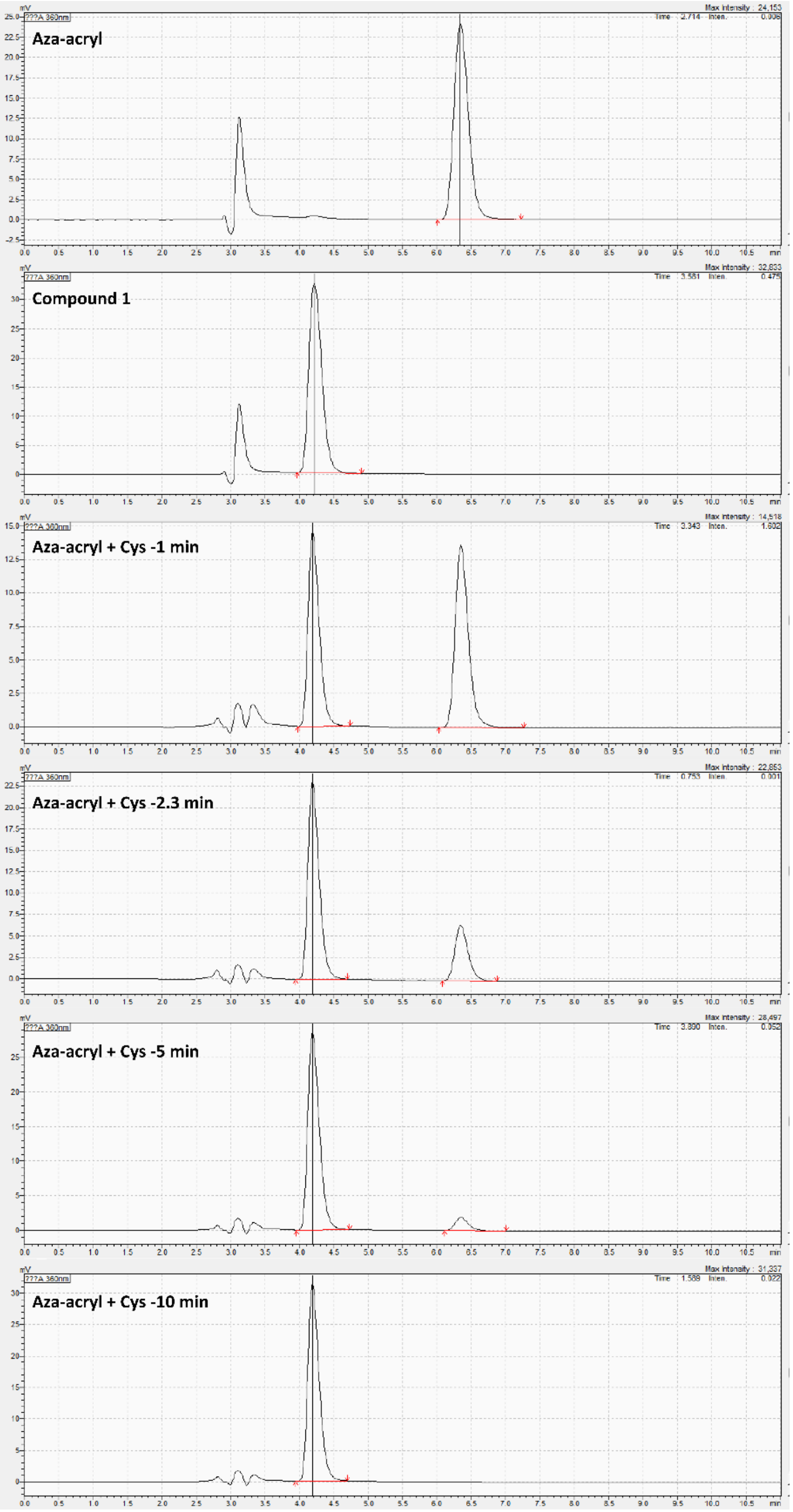

Figure S5. HPLC of Aza-acryl (5 μM in DMSO), Compound 1 (5 μM in DMSO) and the product of Aza-acryl (5 μM) + Cys (10 μM) in EtOH/phosphate buffer (20 mM, pH 7.4; 1:1 v/v) at 1 min, 2.3 min, 5 min, and 10 min. All experiments were conducted at room temperature (r.t.).

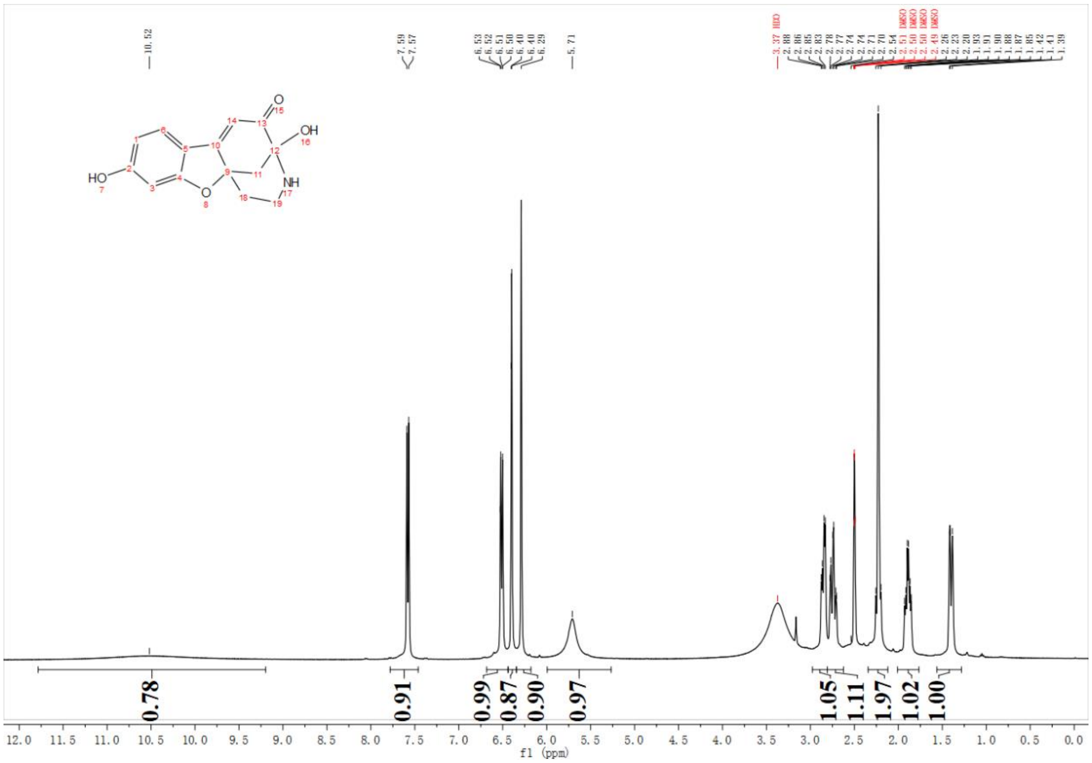

Figure S6. ^{1}H NMR Spectra of Aza (10 mg/mL) in DMSO-d6 at r.t..

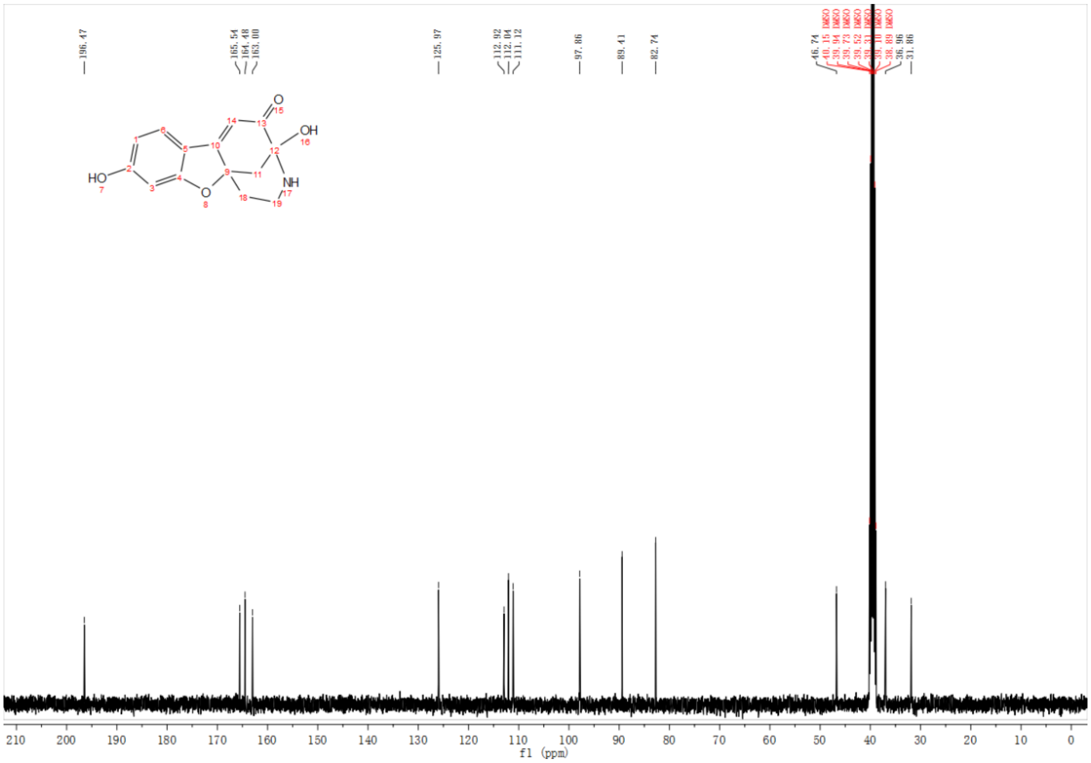

Figure S7. ^{13}C NMR of Aza (10 mg/mL) in DMSO-d6 at r.t..

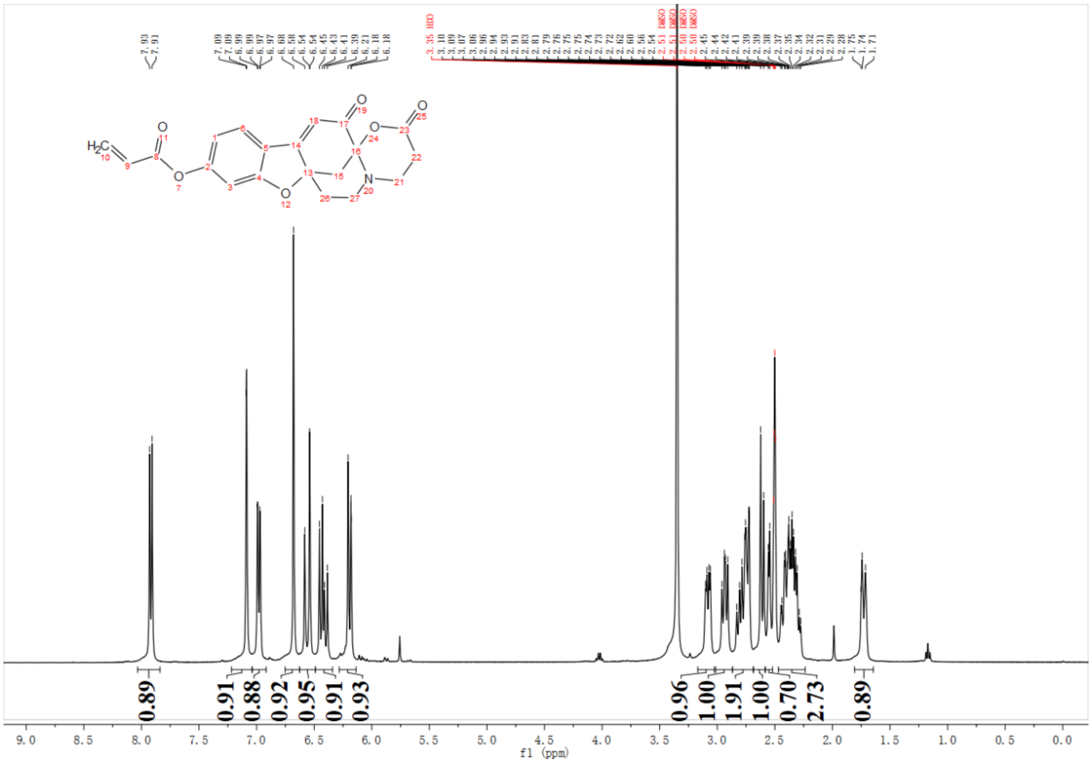

Figure S8. ^{1}H NMR of Aza-acryl (10 mg/mL) in DMSO-d6 at r.t..

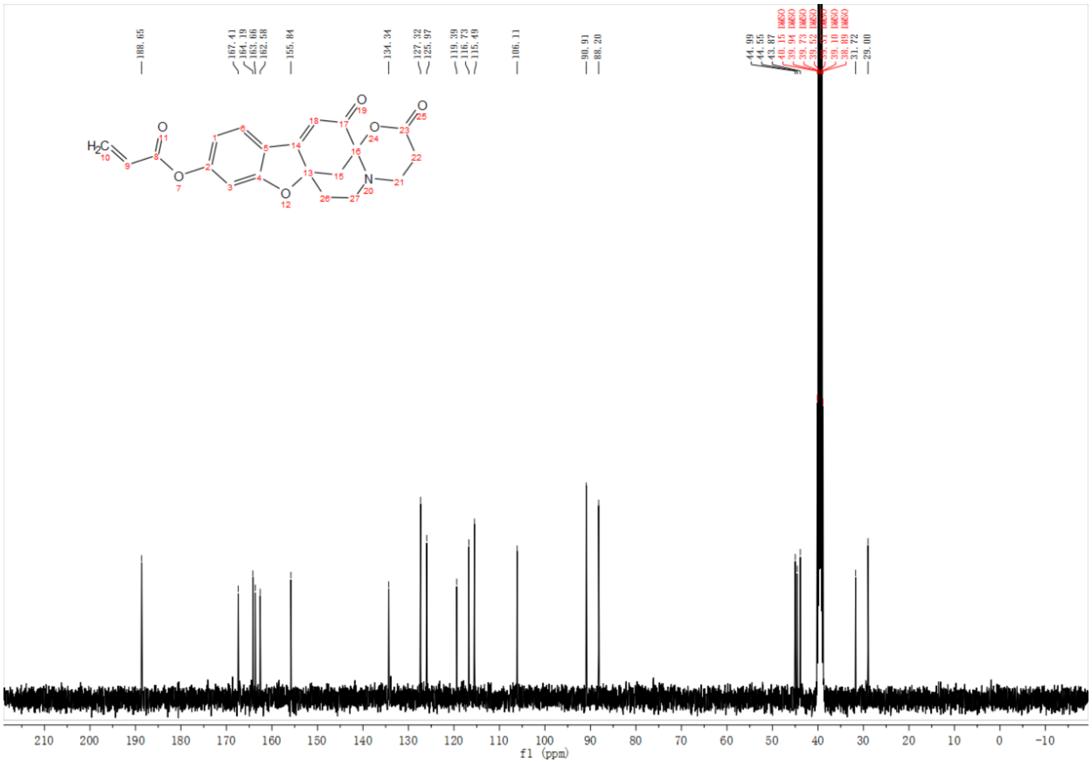

Figure S9. ^{13}C NMR of Aza-acryl (10 mg/mL) in DMSO-d6 at r.t..

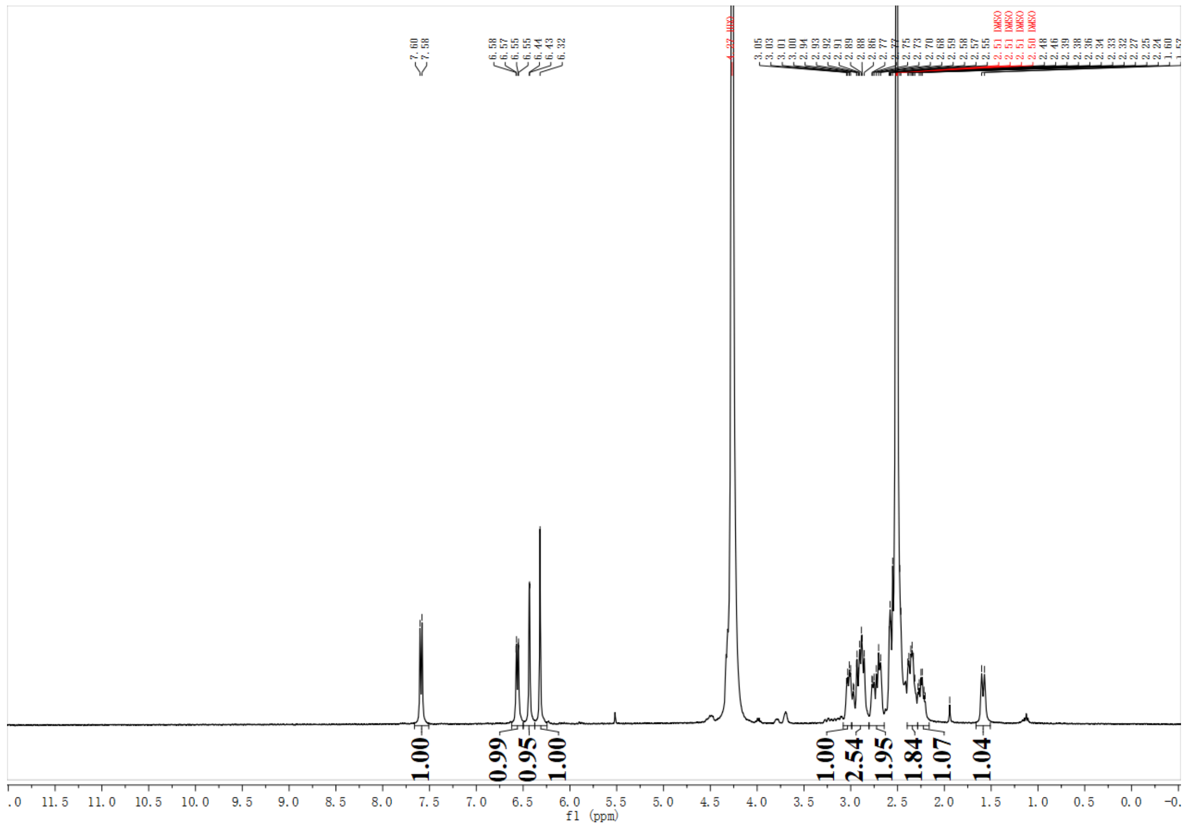

Figure S10. ^1H NMR Spectra of the reaction product of Aza-acryl (10 mg/mL) with Cys (5 mg/mL) and $NaHCO_3$ (0.5 mg/mL) in D_2O/DMSO-d6 =1/1 at r.t..

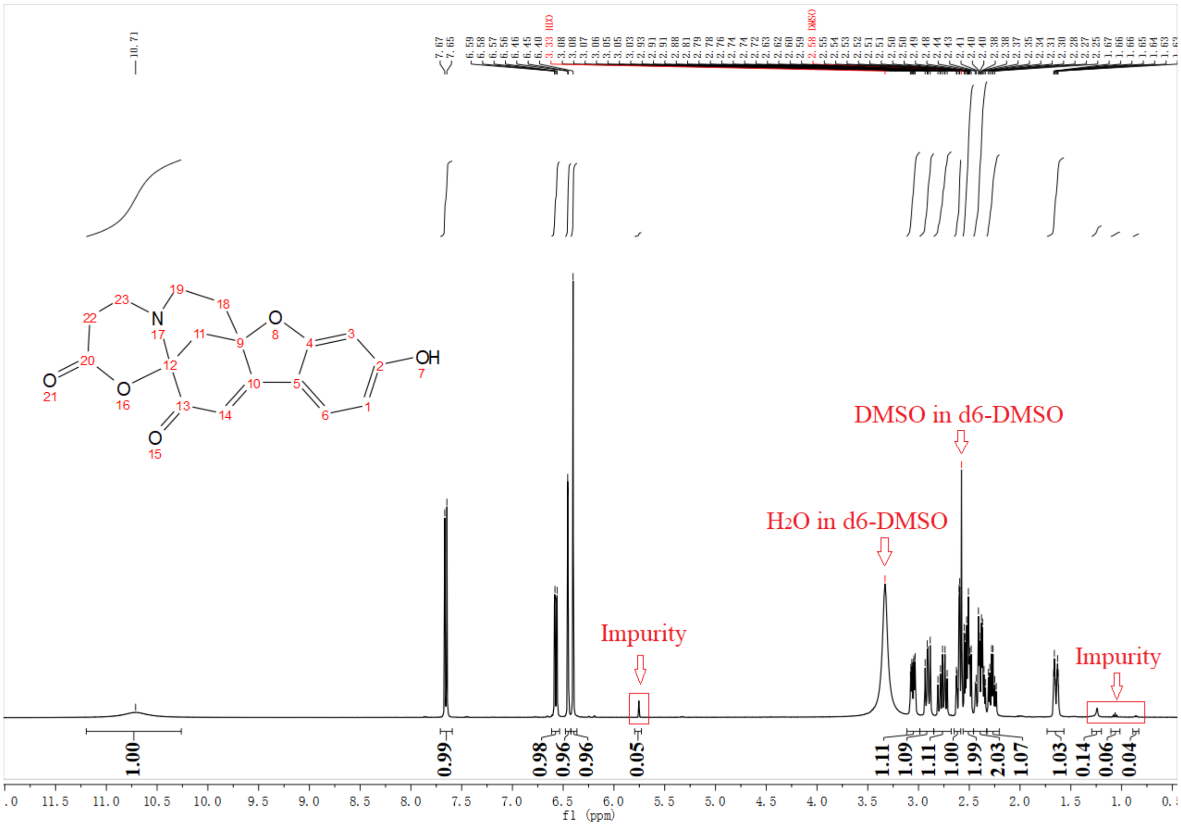

Figure S11. ^{1}H NMR Spectra of Compound 1 (10 mg/mL) in DMSO-d6 at r.t..

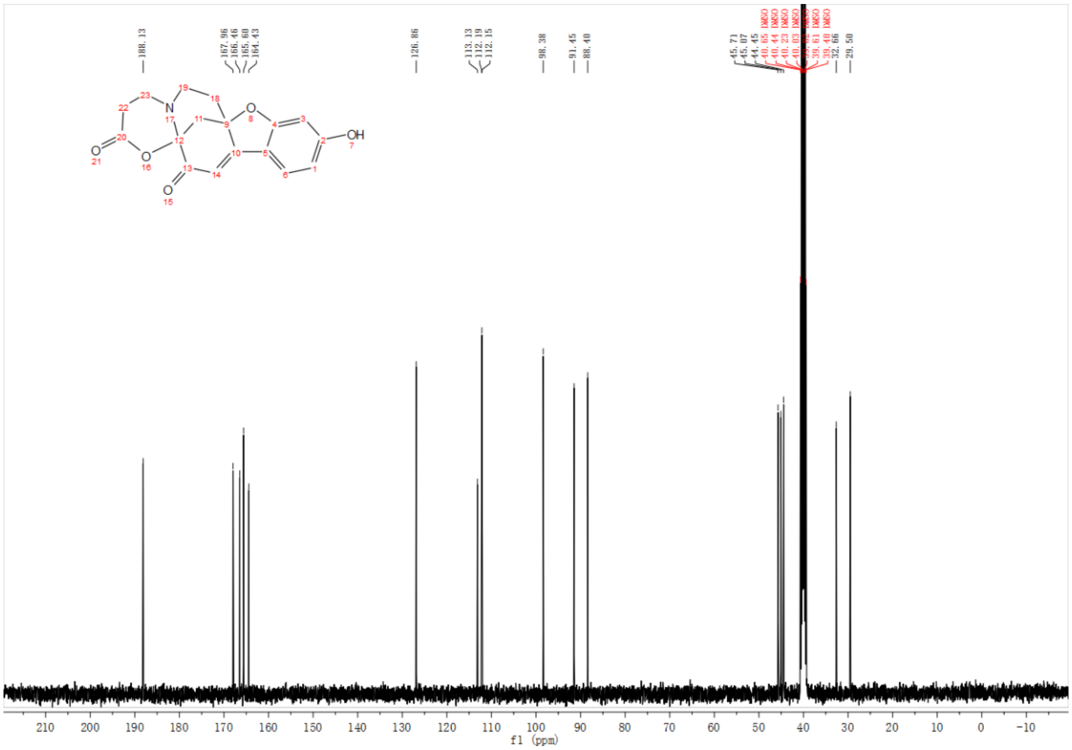

Figure S12. ^{13}C NMR Spectra of Compound 1 (10 mg/mL) in DMSO-d6 at r.t..

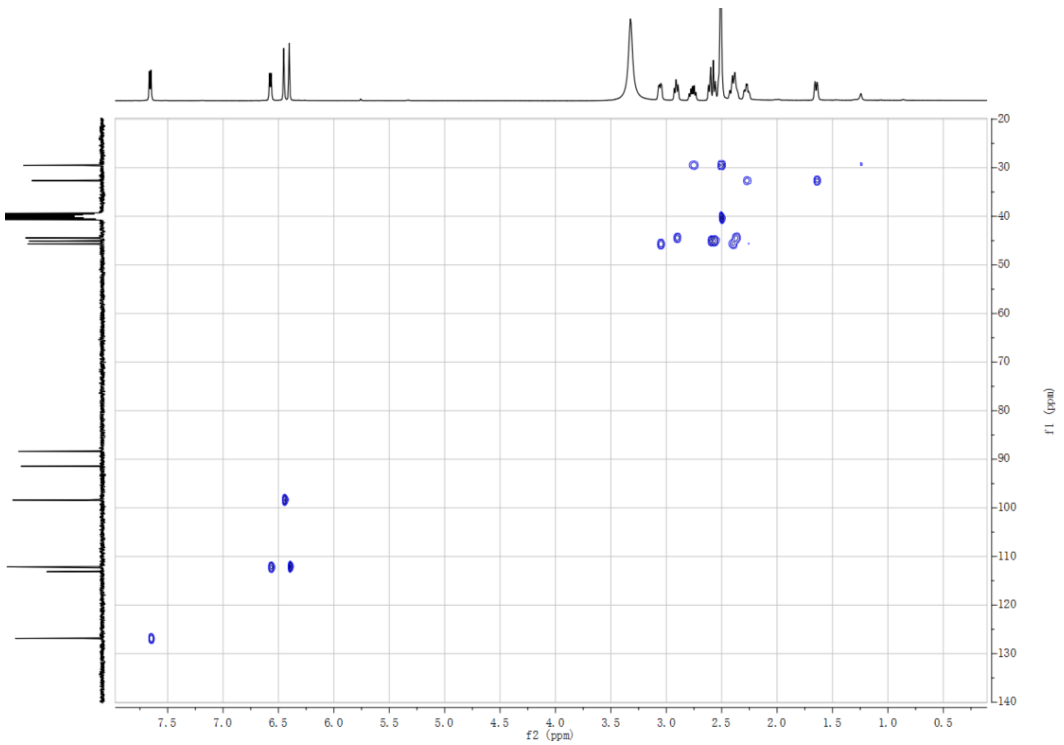

Figure S13. HSQC Spectra of Compound 1 (5 mg/mL) in DMSO-d6 at r.t..

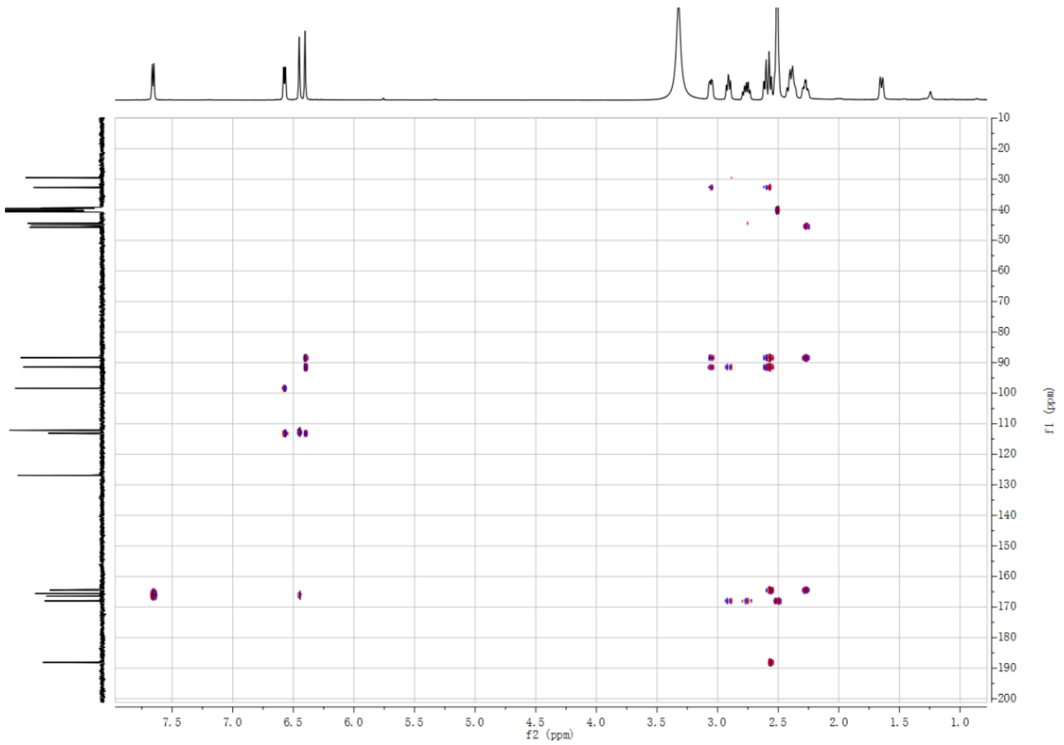

Figure S14. HMBC Spectra of Compound 1 (5 mg/mL) in DMSO-d6 at r.t..

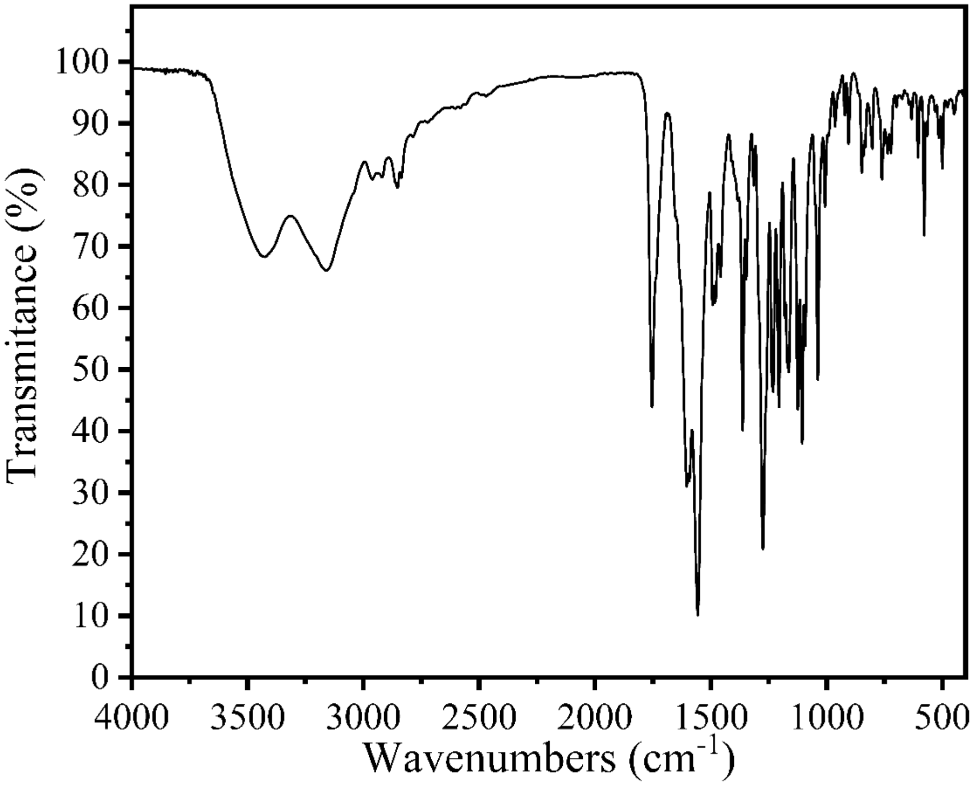

Figure S15. IR Spectra of Compound 1.

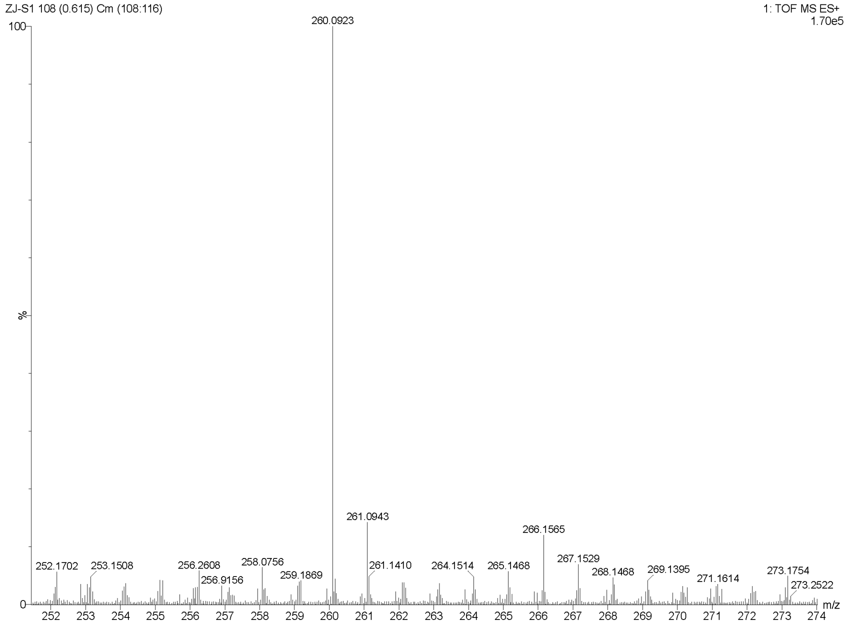

Figure S16. Positive ion ESI mass spectra of the Aza.

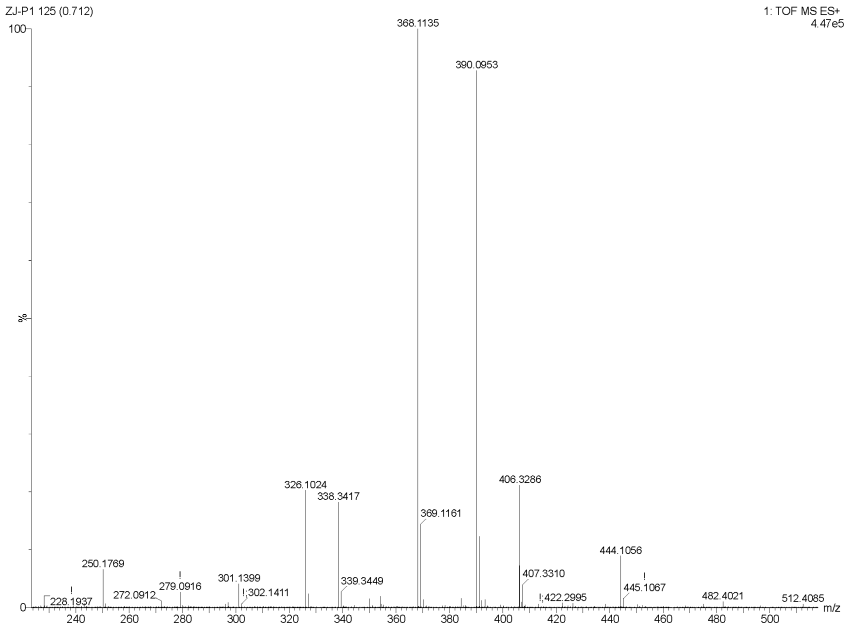

Figure S17. Positive ion ESI mass spectra of the Aza-Aryl

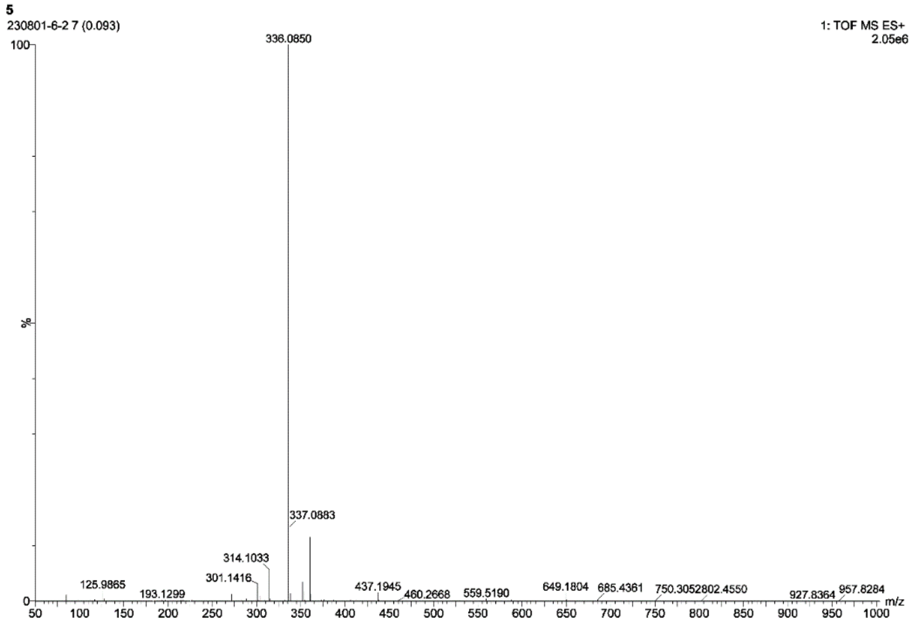

Figure S18. Positive ion ESI mass spectra of Compound 1.

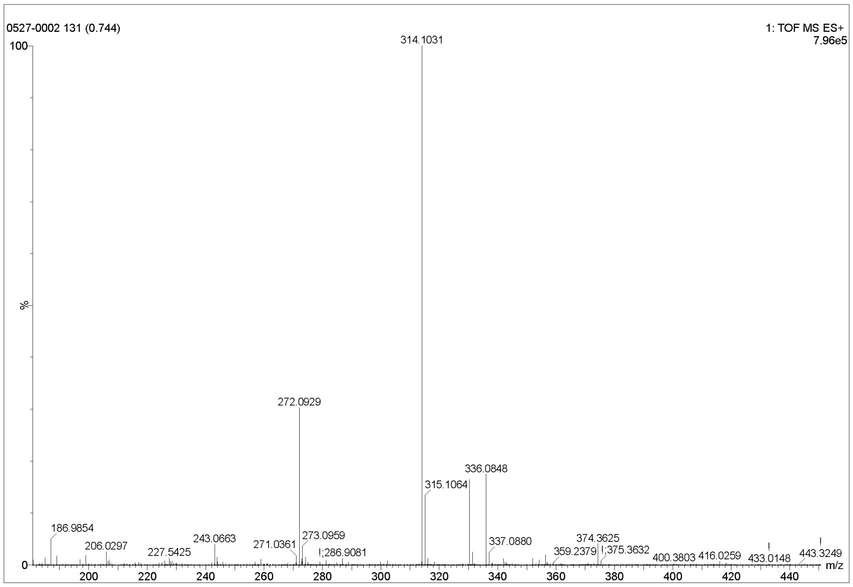

Figure S19. Positive ion ESI mass spectra of Aza-Aryl+Cys.

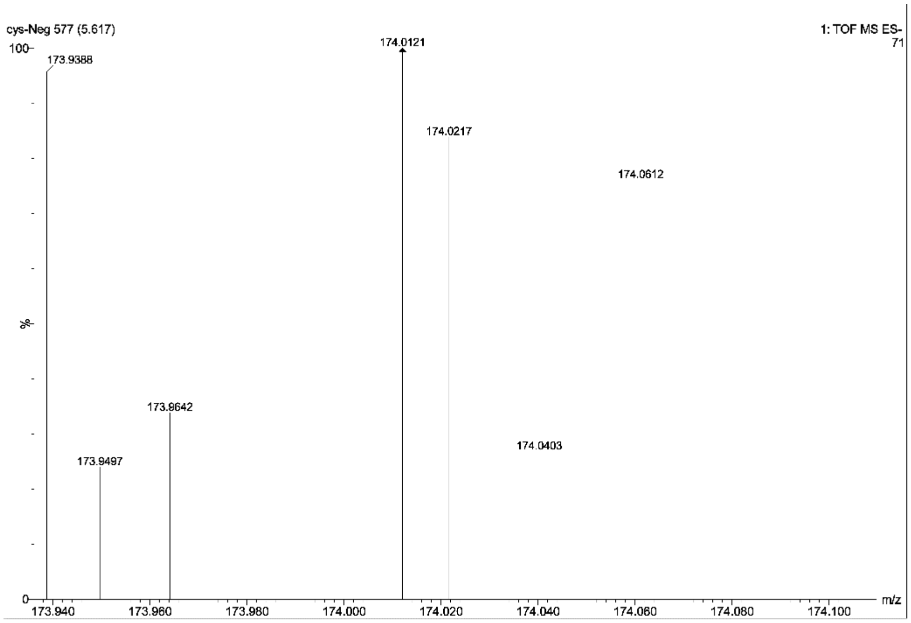

Figure S20. Negtive ion ESI mass spectra of Aza-Aryl+Cys.

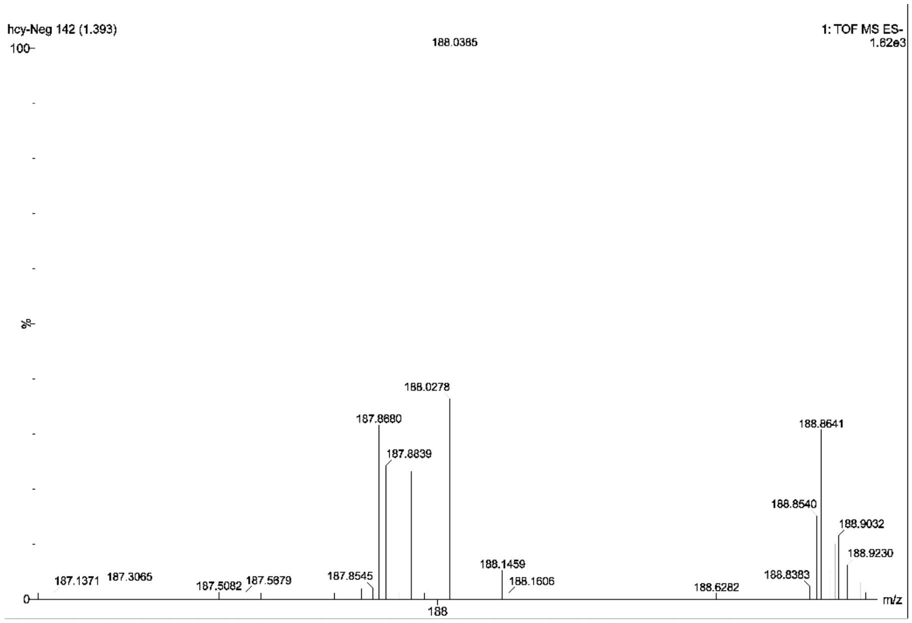

Figure S21. Negtive ion ESI mass spectra of Aza-Aryl+Hcy.

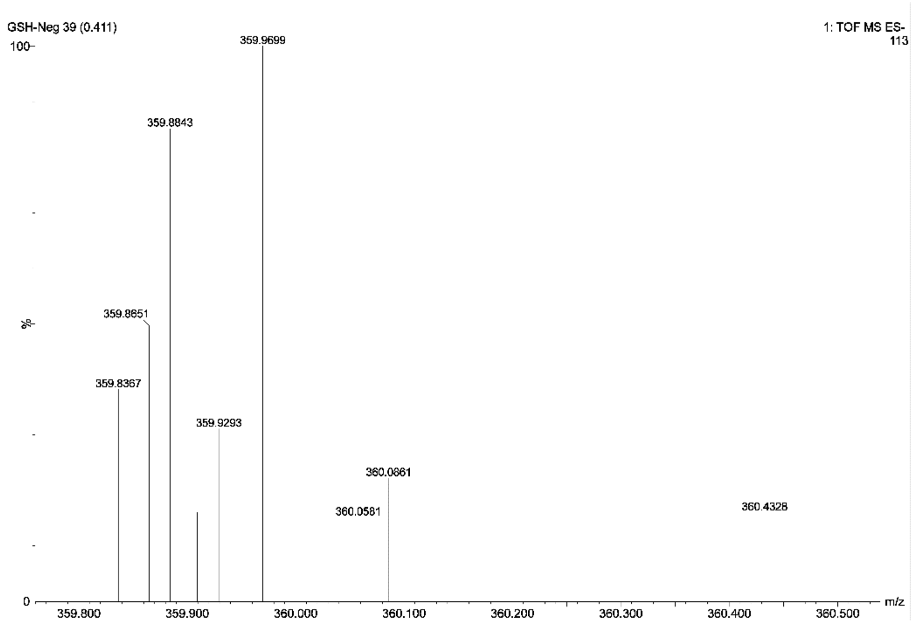

Figure S22 Negtive ion ESI mass spectra of Aza-Aryl+GSH.

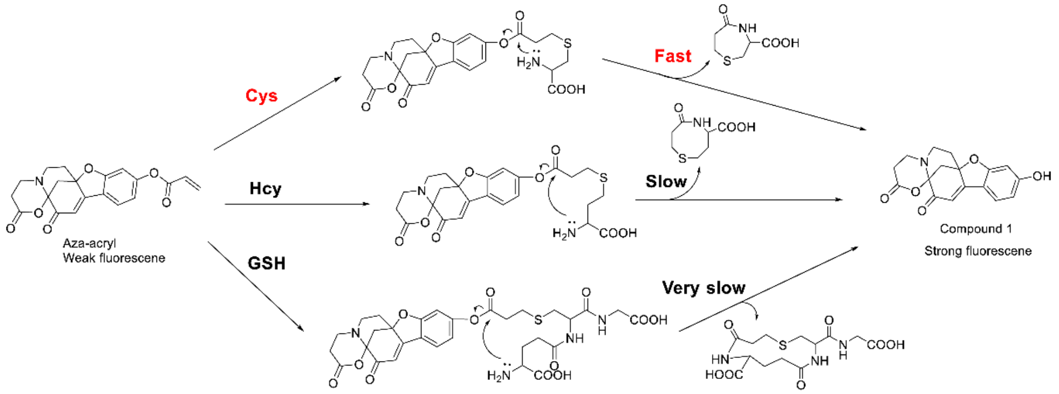

Figure S23 The possible reaction mechanism of Aza-acryl with three biological thiols.

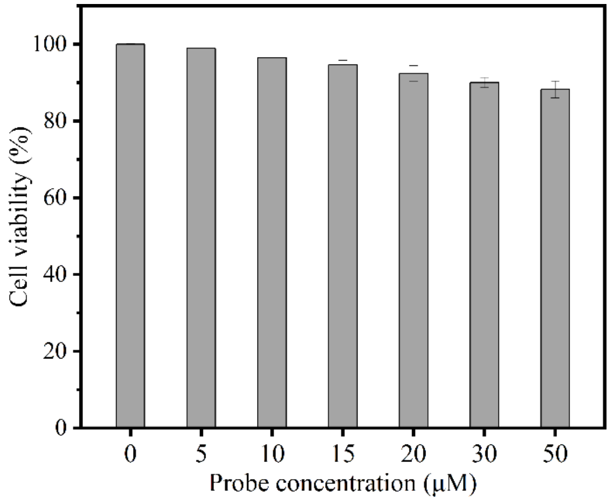

Figure S24. Cytotoxicity of 0-50 μM Aza-Aryl toward A549 cells by CCK-8 assay.

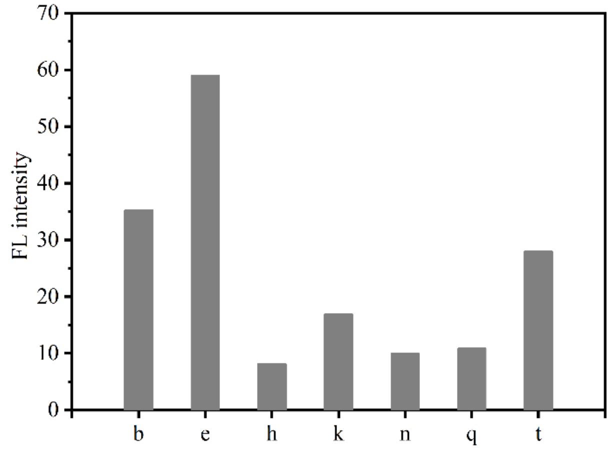

Figure S25. Fluorescence intensities of A549 cells in Figure 8b-t.

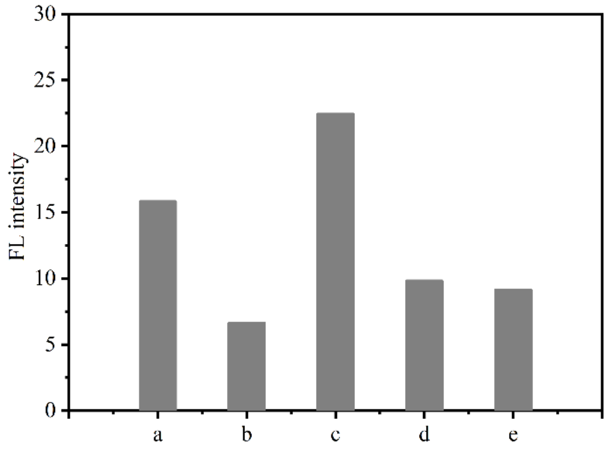

Figure S26. Fluorescence intensities of zebrafishes in Figure 9a-e.

Table S1. Comparisons of linear range and limit of detection (LOD) of previously reported fluorescent probes for detecting Cys.

No.	Structure	Time	Range	LOD	Wavelength	Ref
1	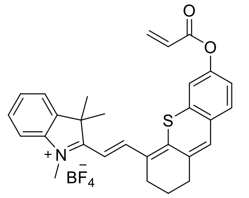	5 min	0-11 μM	21.2 nM	770 nm	[1]
2	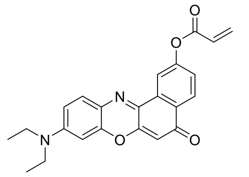	15 min	0–10 μM	19.8 nM	631 nm	[2]
3	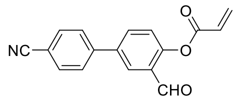	3 min	0–100 μM	3.8 μM	520 nm	[3]
4	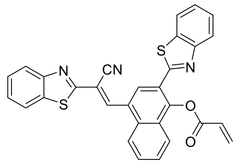	15 min	0–25 μM	76 nM	620 nm	[4]
5	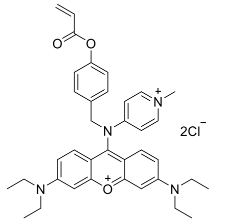	30 min	0–18μM	33.7 nM	540 nm	[5]
6	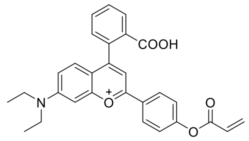	20 min	2–16 μM	44.8 nM	625 nm	[6]

7	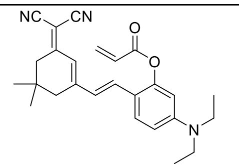	10 min	3-16 μM	1.29 μM	693 nm	[7]
8	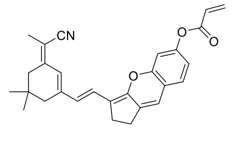	3 min	10.6 nM	73 nM	851 nm	[8]
9	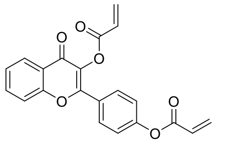	5 min<br>60 min	0–10 μM<br>0-40μM	97 nM<br>97 nM	449 nm<br>509 nm	[9]
10	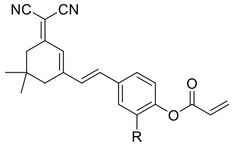	15 min	0-30μM	86.9 nM	666 nm	[10]
11	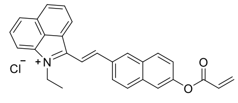	30 min	0-95μM	4 nM	485 nm	[11]
12	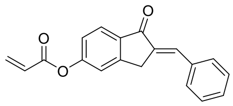	10 min	0-100μM	80 nM	504 nm	[12]
13	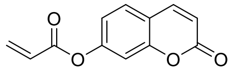	60 min	0-16 μM	65 nM	455 nm	[13]
14	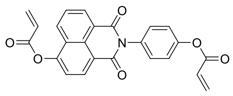	10 min	6-12 μM	0.12 μM	559 nm	[14]
15	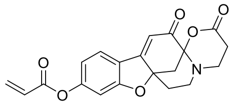	10 min	0.5-4 μM	80 nM	468 nm	This work

Reference

1. Cai, S.; Liu, C.; Jiao, X.; Zhao, L.; Zeng, X. A lysosome-targeted near-infrared fluorescent probe for imaging endogenous cysteine (Cys) in living cells. *Journal of Materials Chemistry B* **2020**, *8*, 2269-2274.
2. Yang, X.-Z.; Wei, X.-R.; Sun, R.; Xu, Y.-J.; Ge, J.-F. Benzoxazine-based fluorescent probes with different auxochrome groups for cysteine detection. *Spectrochimica Acta Part A: Molecular and Biomolecular Spectroscopy* **2020**, *226*, 117582, doi:https://doi.org/10.1016/j.saa.2019.117582.
3. Zhou, B.; Wang, B.; Bai, M.; Dong, M.; Tang, X. Fluorescent probe for highly selective detection of cysteine in living cells. *Spectrochimica Acta Part A: Molecular and Biomolecular Spectroscopy* **2023**, *294*, 122523, doi:https://doi.org/10.1016/j.saa.2023.122523.
4. Zhao, Q.; Qin, J.; Kong, F.; Wang, D.; Guo, Y.; Li, Y. A novel red-emission fluorescent probe for the detection of cysteine in vitro and in vivo. *J. Photochem. Photobiol. A: Chem.* **2023**, *436*, 114383, doi:https://doi.org/10.1016/j.jphotochem.2022.114383.
5. Yang, X.-Z.; Wei, X.-R.; Sun, R.; Xu, Y.-J.; Ge, J.-F. A novel xanthylene-based effective mitochondria-targeting ratiometric cysteine probe and its bioimaging in living cells. *Talanta* **2020**, *209*, 120580, doi:https://doi.org/10.1016/j.talanta.2019.120580.
6. Qiao, L.; Yang, Y.; Cai, J.; Lv, X.; Hao, J.; Li, Y. Long wavelength emission fluorescent probe for highly selective detection of cysteine in living cells. *Spectrochimica Acta Part A: Molecular and Biomolecular Spectroscopy* **2022**, *264*, 120247, doi:https://doi.org/10.1016/j.saa.2021.120247.
7. Liu, H.-B.; Xu, H.; Guo, X.; Xiao, J.; Cai, Z.-H.; Wang, Y.-W.; Peng, Y. A novel near-infrared fluorescent probe based on isophorone for the bioassay of endogenous cysteine. *Organic & Biomolecular Chemistry* **2021**, *19*, 873-877.
8. Chen, Z.; Wang, B.; Liang, Y.; Shi, L.; Cen, X.; Zheng, L.; Liang, E.; Huang, L.; Cheng, K. Near-infrared fluorescent and photoacoustic dual-mode probe for highly sensitive and selective imaging of cysteine in vivo. *Anal. Chem.* **2022**, *94*, 10737-10744.
9. Xu, Z.; Si, S.; Zhang, Z.; Tan, H.; Qin, T.; Wang, Z.; Wang, D.; Wang, L.; Liu, B. A fluorescent probe with dual acrylate sites for discrimination of different concentration ranges of cysteine in living cells. *Anal. Chim. Acta* **2021**, *1176*, 338763, doi:https://doi.org/10.1016/j.aca.2021.338763.
10. Qian, M.; Xia, J.; Zhang, L.; Chen, Q.; Guo, J.; Cui, H.; Kafuti, Y.S.; Wang, J.; Peng, X. Rationally modifying the dicyanoisophorone fluorophore for sensing cysteine in living cells and mice. *Sensors Actuators B: Chem.* **2020**, *321*, 128441, doi:https://doi.org/10.1016/j.snb.2020.128441.
11. Zheng, C.; Zhou, X.; Wang, H.; Ji, M.; Wang, P. A novel ratiometric fluorescent probe for the detection and imaging of cysteine in living cells. *Bioorg. Chem.* **2022**, *127*, 106003, doi:https://doi.org/10.1016/j.bioorg.2022.106003.

12. Zhao, G.; Yang, W.; Li, F.; Deng, Z.; Hu, Y. A turn-on fluorescent probe for real-time detection of endogenous cysteine in living cells. *J. Lumin.* **2020**, *226*, 117506, doi:https://doi.org/10.1016/j.jlumin.2020.117506.
13. Wei, L.-F.; Thirumalaivasan, N.; Liao, Y.-C.; Wu, S.-P. Fluorescent coumarin-based probe for cysteine and homocysteine with live cell application. *Spectrochimica Acta Part A: Molecular and Biomolecular Spectroscopy* **2017**, *183*, 204-208, doi:https://doi.org/10.1016/j.saa.2017.04.051.
14. Yu, Y.; Yang, J.; Xu, X.; Jiang, Y.; Wang, B. A novel fluorescent probe for highly sensitive and selective detection of cysteine and its application in cell imaging. *Sensors Actuators B: Chem.* **2017**, *251*, 902-908, doi:https://doi.org/10.1016/j.snb.2017.05.150.
